# Integrated Multi‐Omics Profiling to Characterize Molecular Subtypes and Reveal Potential Therapeutic Strategies for Colorectal Cancer

**DOI:** 10.1002/mco2.70492

**Published:** 2025-12-08

**Authors:** Xin Guo, Saisai Tian, Xinxing Li, Hongwei Zhang, Anqi Wang, Yan Jin, Ce Bian, Jiayi Lin, Sanhong Liu, Min Tang, Lijun Zhang, Xin Luan, Haiyang Zhou, Weidong Zhang

**Affiliations:** ^1^ Shanghai Frontiers Science Center of Chinese Medicine Chemical Biology Institute of Interdisciplinary Integrative Medicine Research and Shuguang Hospital Shanghai University of Traditional Chinese Medicine Shanghai China; ^2^ School of Pharmacy Shanghai Jiao Tong University Shanghai China; ^3^ School of Pharmacy Naval Medical University Shanghai China; ^4^ Department of Gastrointestinal Surgery Tongji Hospital Tongji University School of Medicine Shanghai China; ^5^ Division of Colorectal Surgery Changzheng Hospital Naval Medical University Shanghai China; ^6^ State Key Laboratory For Quality Ensurance and Sustainable Use of Dao‐di Herbs Institute of Medicinal Plant Development Chinese Academy of Medical Science and Peking Union Medical College Beijing China

**Keywords:** colorectal cancer (CRC), molecular subtype, multi‐omics, therapeutic strategy

## Abstract

Colorectal cancer (CRC) is a complex and heterogeneous disease with limited effective treatment options. To investigate the molecular features and potential therapeutic strategies for CRC patients, including both early‐onset colorectal cancer (EOCRC) and late‐onset colorectal cancer (LOCRC) cases, a comprehensive multi‐omics approach was employed. Whole exome sequencing (WES), RNA sequencing (RNA‐seq), and proteomic and phosphoproteomic profiling were performed on paired tumor and normal adjacent tissue (NAT) from 144 CRC patients, totaling 672 samples. Three distinct molecular subtypes were identified, each exhibiting unique clinical prognoses and molecular characteristics. The S_I subtype was associated with the worst prognosis and a greater prevalence of EOCRC. Moreover, it exhibited a higher stromal score, characterized by increased infiltration of fibroblasts, mesenchymal stem cells, and adipocytes, when compared with the S_II and S_III subtypes. Additionally, the S_II subtype showed a higher immune score. Drug testing using cell lines and patient‐derived three‐dimensional (3D) bioprinted models revealed that S_I tumors were more responsive to Alisertib, suggesting subtype‐specific therapeutic potential. Our study characterized the multi‐omics landscape of CRC, offering critical insights into its molecular heterogeneity. These findings enhance our understanding of the molecular mechanisms underlying CRC and contribute to the development of personalized treatment strategies.

## Graphical abstract

A comprehensive multi‐omics analysis was conducted on Chinese colorectal cancer (CRC) patients, identifying three distinct subtypes. Among these, the S_I subtype exhibited the worst prognosis. This subtype was characterized by a higher prevalence of early‐onset colorectal cancer (EOCRC), an elevated stromal score, and a superior response to Alisertib compared with the other subtypes.

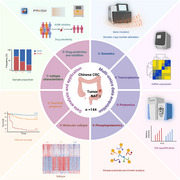

## 1 Introduction

Colorectal cancer (CRC) is the third most common cancer and the second leading cause of cancer‐related deaths worldwide, representing a significant global health burden [1, 2]. Recently, the incidence of CRC has increased, particularly among individuals under 50 years old, a condition known as early‐onset colorectal cancer (EOCRC) [3]. The prognostic outcomes of EOCRC in comparison to late‐onset colorectal cancer (LOCRC) are inconsistent in the literature. Some studies report poorer survival outcomes for EOCRC [4], while others suggest that its prognosis is similar to or better than that of LOCRC [5, 6]. The rising incidence of CRC is attributed to lifestyle factors such as poor diet, physical inactivity, and obesity, as well as environmental exposures and genetic predisposition [7, 8]. Although early detection and treatment options have advanced in recent years, the mechanisms underlying the development of CRC remain complex and not yet fully understood [3, 7].

Currently, the treatment for CRC includes surgery, chemotherapy, and radiotherapy, all of which have contributed to improved survival rates for many patients [9]. However, the tumor heterogeneity among CRC patients, including differences in genetic makeup, molecular profiles, and tumor microenvironment, often makes these standard approaches ineffective for certain individuals [10]. In particular, some patients experience limited response or even resistance to these treatments [10]. As a result, there has been a growing focus on developing targeted therapies and immunotherapies [11, 12, 13], which have shown promise in improving overall survival rates. However, the effectiveness of these newer treatments varies among individuals, with some patients responding favorably while others do not benefit as much [14, 15]. Therefore, understanding the tumor drivers in different CRC patients is crucial for developing personalized therapeutic strategies.

Molecular profiling can help identify the distinct molecular characteristics of different patients [16], and several subtypes have been recognized. Previous genomic studies have identified CRC subtypes [17], but mutational profiling alone has not consistently revealed actionable therapeutic targets. This may be because alterations in the DNA sequence do not necessarily result in changes to gene expression or the corresponding proteins [18]. Moreover, the transcriptomic profiling was used to define the consensus molecular subtypes (CMS) of CRC [19]. However, the relationship between mRNA and protein, as well as their impact on treatment strategies specific to subtypes, is not yet fully comprehended [18]. Although proteomics research has classified five subtypes based on Western CRC cohorts [20], these findings may not apply to Chinese patients. Chen et al. [21] identified three CRC subtypes in a Chinese cohort, but their study was limited to metastatic CRC within the framework of LOCRC. Therefore, it is essential to collect and molecularly classify specimens from EOCRC and LOCRC patients in the Chinese cohort to better understand the molecular mechanisms of CRC and to develop potential individualized therapies.

Multi‐omics analysis holds potential in developing personalized treatment strategies by distinguishing the multilevel molecular characteristics of individual cancer patients [22, 23]. The integrated approach, which combines data from multiple omics layers such as genomics, transcriptomics, proteomics, and phosphoproteomics, has been proven to provide a comprehensive perspective on the mechanisms of various cancers [24, 25, 26]. In particular, mass spectrometry‐based proteomics analysis, when combined with other omics data, can offer valuable biological insights into the occurrence and development of cancers [18, 27]. Therefore, utilizing proteome‐centric data integration to analyze CRC cohorts in China holds promise for understanding the molecular mechanisms and developing personalized treatment strategies.

This study conducted a comprehensive analysis by integrating genomic, transcriptomic, proteomic, and phosphoproteomic data from 144 CRC patients, using paired tumor and normal adjacent tissue (NAT) samples, totaling 672 samples. Our analysis identified three distinct CRC subtypes, each exhibiting significant variations in clinical characteristics. This multi‐omics approach offers crucial insights into the biological mechanisms of CRC, enhancing our understanding of prognosis and aiding in the development of personalized treatment strategies. Additionally, the resulting datasets offer an extensive resource for further CRC research.

## 2 Results

### 2.1 Multi‐Omics Landscape of CRC

**FIGURE 1:**
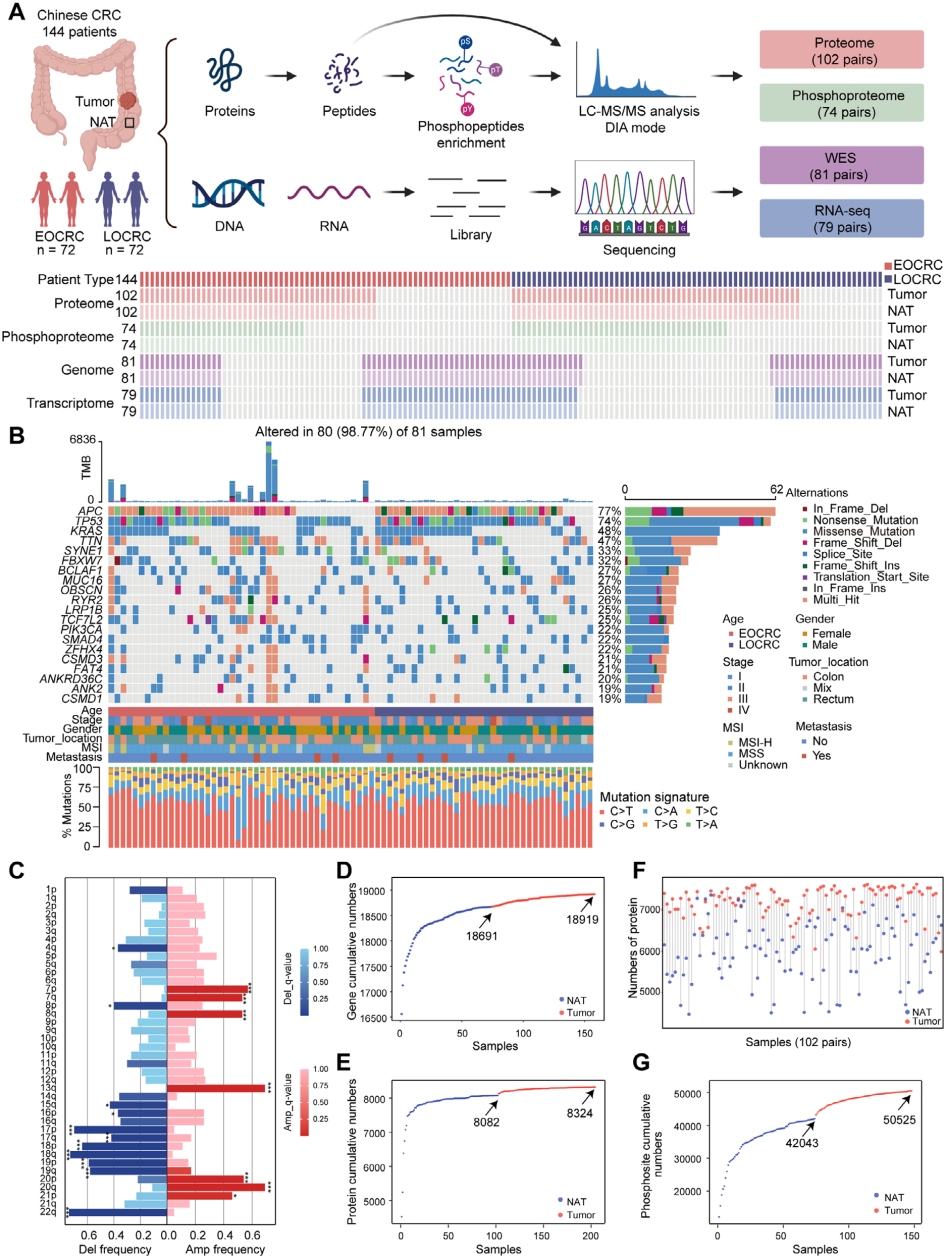
Comprehensive multi‐omics profile of CRC. (A) Schematic of the experimental approach, detailing the sample sizes for multi‐omics analysis, including genomic, transcriptomics, proteomic, and phosphoproteomic. (B) The genomic characteristics of CRC patients are presented, featuring an upper plot of somatic mutations, a middle section emphasizing the most commonly mutated genes, and an adjacent bar plot depicting the distribution of mutation types. The lower panel shows nucleotide substitution proportions. (C) The CRC arm amplification (red) and deletion (blue) landscape. *q*‐value significance levels: *p < 0.05, **p < 0.01, and ***p < 0.001. (D–E) Cumulative gene (D) and protein (E) identifications are presented. (F) Pairwise comparison of proteins is annotated in gray straight lines, and the distribution of protein identifications in tumors (light red) and NATs (light blue). (G) The cumulative number of phosphosite identifications in CRC patients.

We conducted multi‐omics profiling, including whole exome sequencing (WES), RNA sequencing (RNA‐seq), proteome, and phosphoproteome analyses, on paired tumor and NAT samples from 144 Chinese CRC patients at Changzheng Hospital (Shanghai, China) (Figure 1A; Figure S1A; Table S1).

WES of 162 samples revealed APC (77%), TP53 (74%), and KRAS (48%) as the most commonly mutated genes (Figure 1B; Table S2). These findings align with previous studies, further validating the consistency of these mutations across different populations [21]. By conducting somatic copy number alteration (SCNA) analysis (Figure S2A), gains were observed in chromosomes 7p/q, 8q, 13q, 20p/q, and 21p, while the most frequent losses were found in 4q, 8p, 15q, 17p/q, 18p/q, and 22q, etc. (Figure 1C; Table S2). In addition to the previously reported focal alterations at 20q13.33 and 8p11.23 [28, 29], our findings revealed notable amplifications in 12p13.32 and 6p21.1, as well as deletions in 18q23 and 4p16.3 (Figure S2B,C; Table S2). Moreover, RNA‐seq of 158 samples (79 paired tumors and NATs) identified 18,919 genes (Figure 1D; Figure S1C), with all samples passing quality control (Figure S1B). Additionally, we characterized 8324 proteins from 204 samples and 50,525 phosphosites from 148 samples using data‐independent acquisition (DIA) methods (Figure 1E–G; Figure S1D–F). More genes, proteins, and phosphosites were identified in tumor tissues compared with NATs (Figure 1D–G). Hybrid spectral libraries for the CRC proteome and phosphoproteome were generated using the Proteome Discoverer and Spectronaut platforms. In total, the CRC proteome spectral library comprised 229,103 precursors, 154,756 peptides, and 12,493 protein groups. The CRC phosphoproteome spectral library included 248,055 phosphoprecursors, 113,529 phosphopeptides, and 11,446 phosphoprotein groups.

Quality control using 293T cells showed high reproducibility, with average Spearman's correlation coefficients of 0.96 and 0.93 for proteomic and phosphoproteomic standards, respectively (Figure S1G–H; Table S8). Principal component analysis (PCA) and partial least squares discrimination analysis (PLS‐DA) demonstrated distinct differences between tumors and NATs at the transcriptomic (Figure S3A,B), proteomic (Figure S3C,D), and phosphoproteomic levels (Figure S3E,F). Overall, our comprehensive multi‐omics analysis provides an in‐depth molecular characterization of CRC.

### 2.2 Subtype Classification of CRC Based on Proteomic Data

**FIGURE 2:**
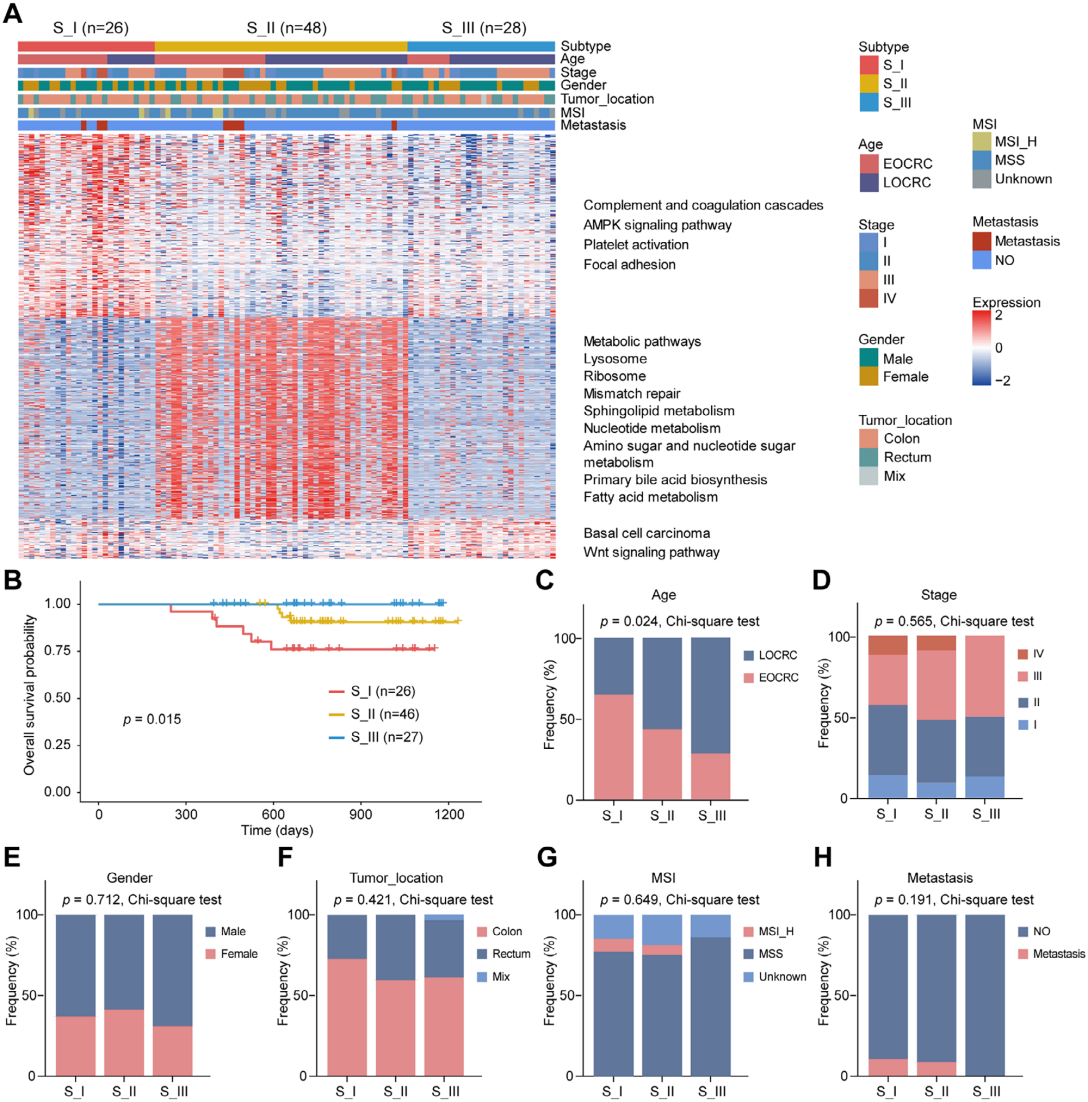
Proteomic subtyping of CRC and its associations with clinical outcomes. (A) Consensus clustering analysis was conducted on differentially expressed proteins between tumor tissues and NATs, with columns representing patient samples and rows representing proteins. (B) Kaplan–Meier survival curves categorized by proteomic subgroups, accompanied by log‐rank test *p*‐values. (C–H) Distribution of clinical characteristics, including age (C), tumor stage (D), gender (E), tumor location (F), MSI status (G), and metastasis (H) across proteomic subtypes, with significance assessed using the chi‐square test.

The incidence of EOCRC, defined as cases occurring in individuals under the age of 50, has been increasing annually [3]. No significant prognostic differences were observed between EOCRC and LOCRC in our cohort, as well as in the CPTAC (Clinical Proteomic Tumor Analysis Consortium) cohort (Figure S4A,B). The differential proteins and enriched signaling pathways between EOCRC and LOCRC are presented in Figure S4C,D. The results indicated that there were 692 differential proteins between EOCRC and LOCRC (fold change > 1.2, *p*‐value < 0.05, Figure S4C). Compared with LOCRC, the significantly upregulated signaling pathways in EOCRC primarily included carbon metabolism, glycolysis, gluconeogenesis, and biosynthesis of amino acids, while the significantly downregulated pathways mainly involved ATP‐dependent chromatin remodeling, ribosome biogenesis in eukaryotes, and nucleocytoplasmic transport (Figure S4D). Using proteomic data, we characterized the molecular heterogeneity of CRC patients by selecting the 1035 most variable proteins for consensus clustering analysis (Figure S5A–H; Table S6). This analysis identified three distinct subtypes: S_I (26 samples), S_II (48 samples), and S_III (28 samples) (Figure 2A).

Significant differences in overall survival times were observed among the subtypes, with the S_I subtype showing the worst prognosis (*p* = 0.015) (Figure 2B). The CRC subtypes remained an independent prognostic factor when adjusted for other clinicopathological characteristics in multivariate Cox regression analysis (*p* < 0.001, Figure S5I). Proteomic analysis identified distinct differences in the S_I subtype compared with the S_II and S_III subtypes (Figure 2A). Proteins exhibiting differential expression were identified based on a fold change >2 and an adjusted *p*‐value < 0.05, allowing for the enrichment of crucial molecular pathways associated with each CRC subtype. The S_I subtype showed increased activity in complement and coagulation cascades, AMPK signaling, platelet activation, and focal adhesion (Table S6). In addition, the S_II subtype exhibited notable enrichment in pathways related to metabolic processes, such as nucleotide metabolism, fatty acid metabolism, sphingolipid metabolism, primary bile acid biosynthesis, and mismatch repair (Table S6). The characteristic signaling pathways were also observed in an independent single‐cell transcriptomics dataset (GSE132465, Figure S6F). Moreover, the S_III subtype was characterized by enrichment in the Wnt signaling pathway (Table S6).

Further statistical analysis was conducted to compare the clinicopathologic characteristics among the three subtypes, including age, tumor stage, gender, tumor location, MSI (microsatellite instability), and metastasis (Figure 2C–H; Table S6). The analysis revealed no significant differences among the subtypes in terms of clinicopathologic characteristics, except for age (*p* = 0.024, Chi‐square test; Figure 2C). In the S_I subtype, EOCRC cases were more frequent than LOCRC cases, while in the S_III subtype, LOCRC cases exceeded EOCRC cases (Figure 2C). In the S_II subtype, EOCRC and LOCRC occur in approximately equal numbers (Figure 2C). None of the CRC patients with S_III subtype developed metastasis, which may partly explain the best prognosis for this subtype (Figure 2H).

Overall, our comprehensive proteomic profiling identified three distinct subtypes of CRC, each characterized by unique molecular profiles and associated clinical characteristics. These findings pave the way for more personalized treatment strategies, offering the potential for improved patient outcomes by tailoring therapies to the specific molecular characteristics of each CRC subtype.

### 2.3 Proteomic Characteristics Among Three CRC Subtypes

**FIGURE 3:**
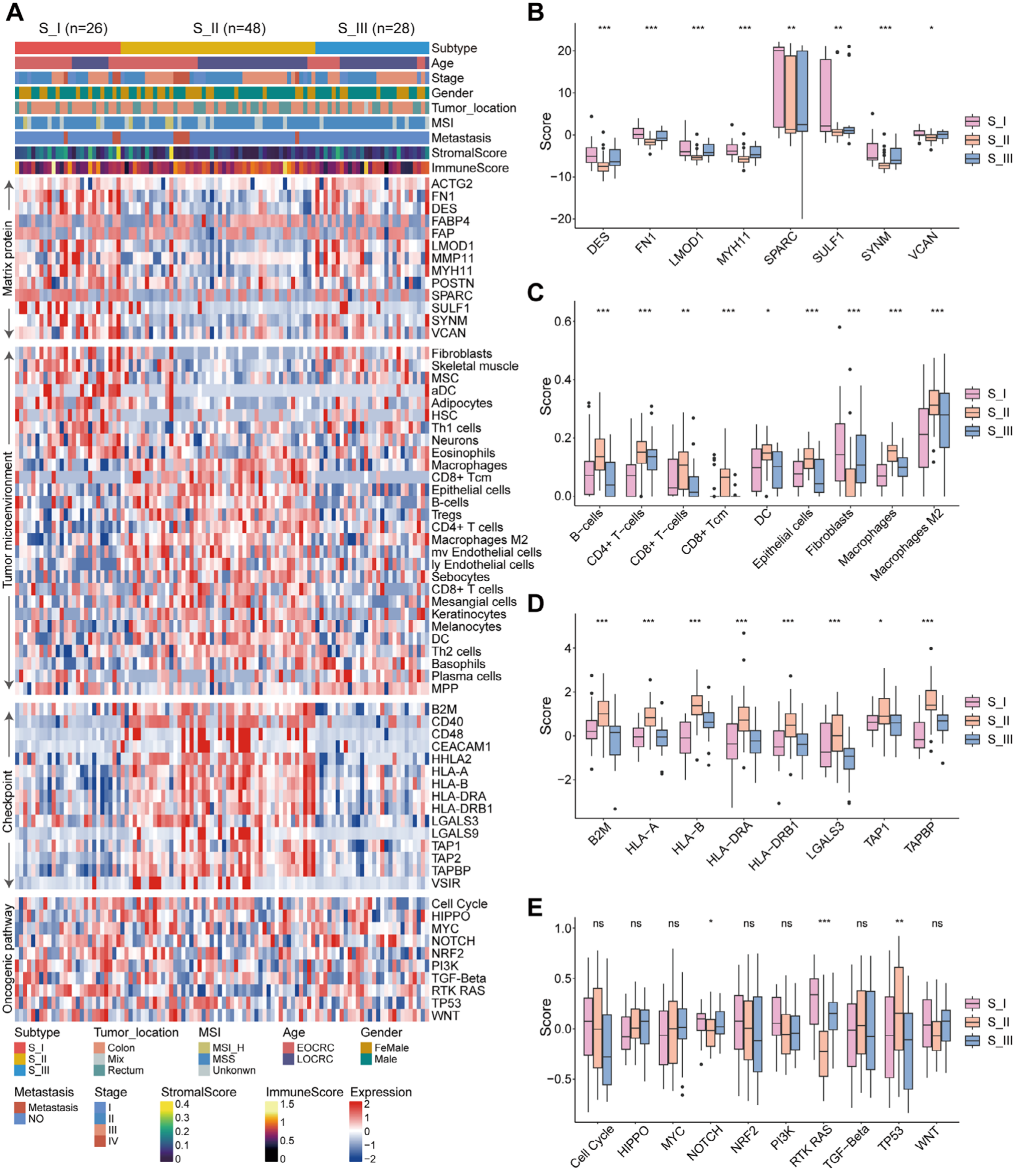
Proteomic characteristics of three subtypes. (A) The upper heatmap displays the clinical characteristics (top panel) alongside the xCell signature scores. The lower heatmap illustrates matrix proteins, microenvironmental cells, immune checkpoints, and oncogenic pathways across the three proteomic subtypes. (B–E) Boxplots depicting the abundance of representative matrix proteins (B), microenvironmental cells (C), immune checkpoints (D), and oncogenic pathways (E) among different proteomic subtypes (Kruskal–Wallis test).

We further examined the proteomic characteristics of different CRC subtypes. Using the xCell algorithm, we calculated immune infiltration across the three subtypes at the proteomics level. The results indicated that the S_I subtype exhibited an elevated stromal score, whereas the S_II subtype demonstrated a higher immune score (Figure 3A; Figure S6A,B; Table S9). The findings were also observed in an independent single‐cell transcriptomics dataset (GSE132465, Figure S6C–E). The S_I subtype exhibited high expression levels of several stromal genes, such as DES, FN1, LMOD1, MYH11, SPARC, SULF1, SYNM, and VCAN (Figure 3A,B). Meanwhile, the S_I subtype showed greater infiltration of fibroblasts, mesenchymal stem cells, and adipocytes, compared with those of the other subtypes (Figure 3A; Table S9). Immunohistochemical (IHC) results also indicated that the fibroblast marker α‐SMA was more highly expressed in the S_I subtype (Figure S7A). Cancer‐associated fibroblasts are crucial in CRC progression and poor prognosis [30], potentially contributing to the worse prognosis in S_I subtype patients.

The S_I and S_III subtypes exhibited immune “cold” characteristics, marked by minimal immune cell presence and a noninflamed tumor microenvironment. In contrast, the S_II subtype was immune “hot”, exhibiting higher levels of immune cells (CD8^+^ T, CD4^+^ T, B cells, dendritic cells, and macrophages) (Figure 3C; Table S9), along with elevated expression of immune checkpoints (B2M, HLA‐A, HLA‐B, LGALS3, TAPBP; *p* < 0.05; Figure 3D). The IHC results also indicated that CD4 (T helper cells), CD8 (cytotoxic T cells), CD19 (B cells), and CD68 (macrophages) were more highly expressed in the S_II subtype (Figure S7B–E). The increased expression of these markers in S_II tumors further supports that this subtype may benefit from immunotherapy strategies aimed at enhancing the immune response. Gene set variation analysis identified activation of the NOTCH and RTK‐RAS pathways in the S_I subtype (*p* < 0.05; Figure 3E). Hence, our analysis displays distinct proteomic and immune characteristics across CRC subtypes, providing insights into their molecular heterogeneity.

The S_I subtype is notably associated with the most unfavorable clinical outcomes (Figure 2B). To identify signature proteins associated with this aggressive subtype, we selected proteins that exhibited subtype‐specific high expression patterns (Figure S8A). The expression levels of 18 signature proteins in the S_I subtype were significantly higher compared with the other subtypes, and high expression of these proteins was correlated with poor prognosis. In the multivariate Cox regression analysis, EIF4A1 emerged as the most unfavorable risk factor based on the risk score (Figure S8B). Therefore, we further investigated whether EIF4A1 impacted the aggressive phenotype of CRC. First, EIF4A1 was efficiently knocked down in HCT116 cells by siRNA transfection, achieving a knockdown efficiency of over 70% (*p* < 0.001, Figure S8D). The crystal violet staining indicated that the knockdown of EIF4A1 could significantly inhibit CRC cell proliferation (*p* < 0.01, Figure S8C; Figure S8E). Furthermore, transwell assays revealed that the migration ability of HCT116 cells was also suppressed following EIF4A1 knockdown (*p* < 0.01; Figure S8F–G). In conclusion, our analysis revealed that high EIF4A1 expression is associated with poor prognosis in CRC patients, and its knockdown reduces proliferation and migration in CRC cells.

### 2.4 Genomic and Transcriptomics Differences Among Different CRC Subtypes

**FIGURE 4:**
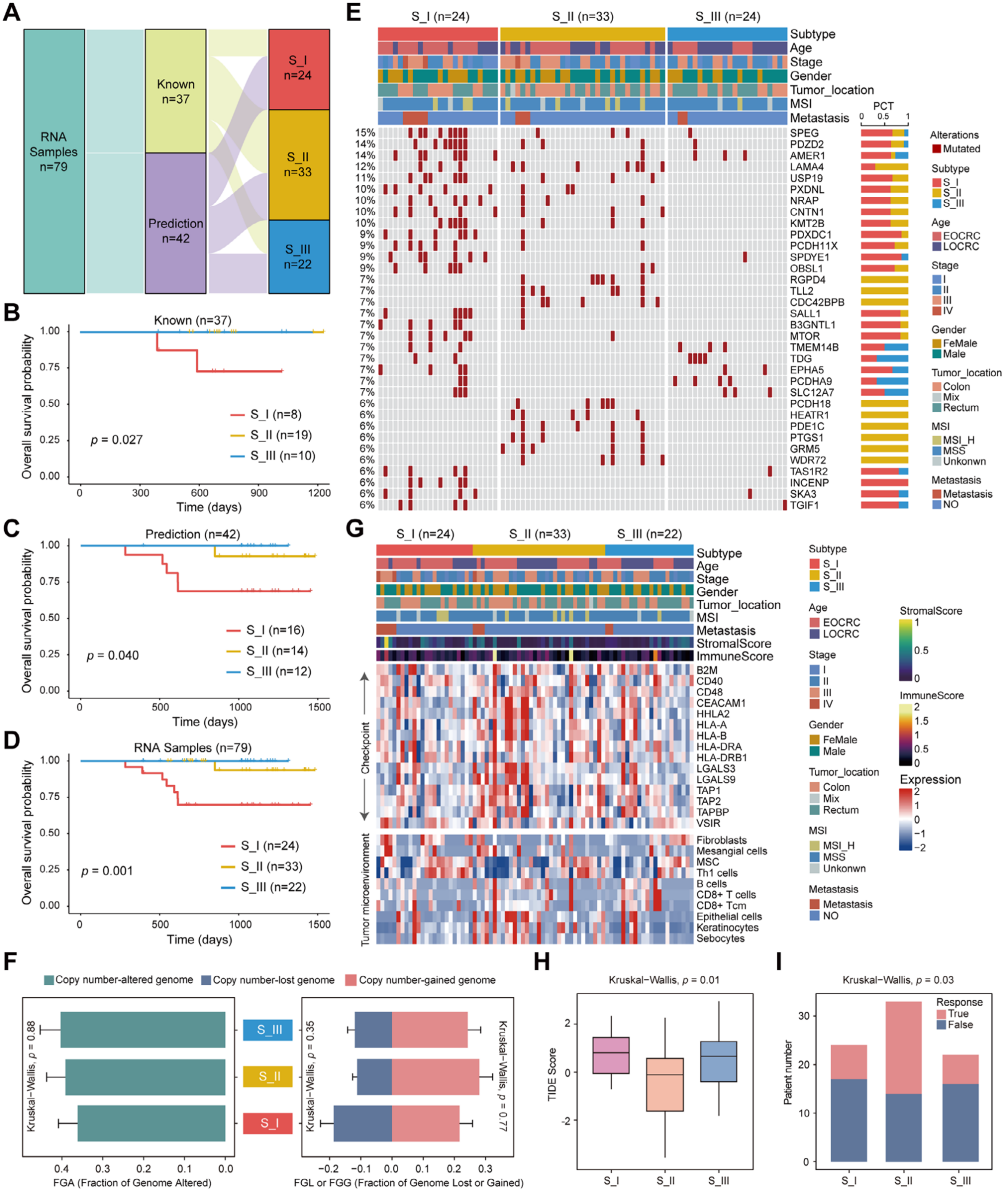
Genomic and transcriptomic features analyses across different proteomic subtypes. (A) Analysis of transcriptome data features for subtype classification of unknown samples. (B–D) Kaplan–Meier survival curves illustrate overall survival for the three proteomic subtypes in known (B), predicted (C), and combined transcriptomic samples (D). (E) The genomic profile of CRC patients is shown, with the upper heatmap indicating clinical features, the middle panel presenting the top 34 differentially mutated genes, and the bar chart on the right illustrating gene‐mutation distribution across subtypes. (F) Bar charts show the mean FGA and FGL/FGG values with standard error. (G) The upper heatmap displays clinical features and xCell signature scores, while the lower heatmap shows immune checkpoint and microenvironmental cell distribution across the three subtypes. (H) TIDE scores across subtypes were analyzed using the Kruskal–Wallis test. (I) Immune response analysis across subtypes reveals differences in the immune landscape.

As depicted in Figure 1A, there is a discrepancy in sample distribution between RNA‐seq and proteome sequencing, with 37 samples matched one‐to‐one. Consequently, the classification of these 37 samples could be clearly defined based on proteomic subtypes, whereas the classification of the remaining 42 samples remained unknown (Figure 4A). To classify each of these remaining samples into CRC subtypes, the top 30 most differentially expressed genes were first identified and sorted by log_2_(fold change) as subtype‐specific genes at the mRNA level for each CRC subtype (Table S10). Subsequently, the remaining samples were subjected to nearest template prediction (NTP) [31] analysis to determine their subtypes. The remaining samples were distributed into the following groups: 16 samples in the S_I subtype, 14 samples in the S_II subtype, and 12 samples in the S_III subtype (Figure S9A). In the full cohort, there were 24 samples in the S_I subtype, 33 samples in the S_II subtype, and 22 samples in the S_III subtype (Figure 4A). Survival analysis indicated significant prognostic differences among the three subtypes at the transcriptome level (*p* < 0.05; Figure 4B–D), with patients in the S_I subtype exhibiting the poorest outcomes.

Next, the genomic heterogeneity of the three CRC subtypes was examined, and the S_I subtype had the most mutations among the commonly altered genes (Figure 4E; Table S11). No statistical difference was found in the fraction of genome altered (FGA), including fraction of genome losses (FGL) and fraction of genome gains (FGG), across the three CRC subtypes (Figure 4F). Given the immune‐hot phenotype of the S_II subtype observed at the proteomics level, the same approach was applied at the transcriptome level to assess immune cell abundance for each sample. The S_II subtype exhibited an increased presence of immune cells, including B cells and CD8^+^ T cells, along with elevated expression of immune checkpoint molecules (Figure 4G). Transcriptomic analysis using tumor immune dysfunction and exclusion (TIDE) prediction indicated that the S_I subtype exhibited significantly elevated TIDE scores (Figure 4H), indicating stronger immune evasion. Additionally, a higher proportion of cases within the S_I subtype were predicted to be nonresponders to immunotherapy (Figure 4I). Therefore, it was hypothesized that the proposed S_I subtype is resistant to immunotherapy. In contrast, the S_II subtype showed significantly lower TIDE scores, with more cases predicted to be responders, indicating that the S_II subtype is more suitable for immunotherapy (Figure 4H,I).

Our results indicate that the different subtypes of CRC exhibit distinct genomic and transcriptomic profiles, significantly highlighting their unique molecular characteristics. These variations suggest underlying differences in the pathological mechanisms driving tumor development and progression across subtypes. Moreover, these findings emphasize the potential for personalized treatment strategies tailored to each CRC subtype, offering the possibility of more effective therapies that could improve patient outcomes.

### 2.5 Phosphoproteomic Characterization of CRC

**FIGURE 5:**
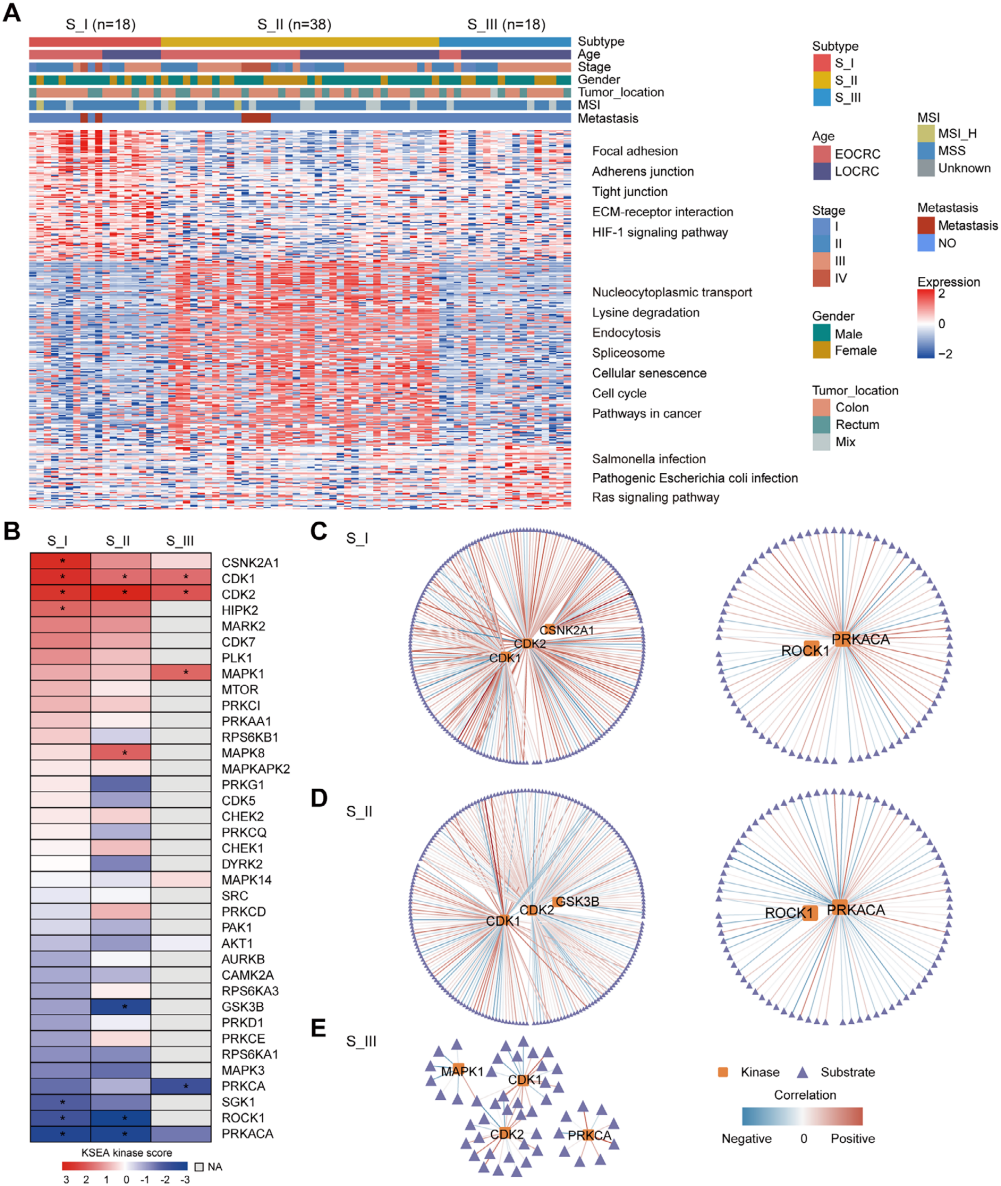
Comparative analysis of phosphoproteomic characteristics among the three proteomic subtypes. (A) A heatmap depicting phosphoproteomic data according to the proteomic classification, with each column representing an individual patient sample and each row corresponding to a phosphoprotein. (B) Enrichment analysis of kinases associated with differentially expressed phosphosites in each proteomic subtype (Fisher's exact test, *p < 0.05). (C–E) Kinase–phosphosubstrate regulatory networks for the S_I (C), S_II (D), and S_III (E) subtypes.

Phosphorylation modifications in CRC patients were systematically studied to better understand the molecular mechanisms driving tumor progression and heterogeneity. A thorough analysis revealed distinct phosphoprotein expression patterns among the three proteomic subtypes of CRC (Figure 5A; Table S12). In the S_I subtype, differential protein phosphorylation was notably enriched in pathways related to focal adhesion, adherens junction, tight junction, extracellular matrix (ECM)‐receptor interaction, and HIF‐1 signaling pathways (Figure 5A; Table S12). These pathways are crucial for cellular adhesion, migration, and survival under hypoxic conditions, which are often associated with aggressive tumor behavior [32, 33]. In addition, the S_II subtype displayed a significant enrichment in pathways related to nucleocytoplasmic transport and cell cycle regulation (Figure 5A; Table S12). Overall, the different phosphorylation patterns of these CRC subtypes highlight their unique molecular characteristics and provide potential targets for subtype‐specific therapeutic interventions.

Based on the differential phosphorylation sites observed across the CRC subtypes, we further investigated the corresponding kinase activities (Figure 5B; Figure S9B–D). Each subtype exhibited distinct kinase enrichment, with varying levels of kinase activity observed across the different subtypes (Figure 5B). The S_I subtype was characterized by the highest number of activated kinases, resulting in an elevated kinase‐substrate enrichment analysis (KSEA) kinase score relative to the other subtypes (Figure 5B). It suggested that a more active kinase network in this subtype potentially drives its malignant tumor behavior. CDK1 and CDK2, crucial regulators of cell cycle progression [34, 35, 36], were significantly enriched across all subtypes, highlighting their critical role in CRC pathogenesis (Figure 5B). The S_I subtype exhibited a specific enrichment of CSNK2A1 and HIPK2 (Figure 5B), both involved in regulating cellular processes such as transcription, DNA repair, and apoptosis [37, 38]. MAPK8 was predominantly enriched in the S_II subtype, while MAPK1 exhibited distinct enrichment in the S_III subtype (Figure 5B).

To further explore the complexity of kinase activity and its downstream effects, we constructed kinase–substrate regulatory networks for each of the CRC proteomic subtypes (Figure 5C–E; Table S12). The S_I subtype displayed the most complex regulatory network, with a greater number of kinases interacting with a larger pool of phosphosubstrates (Figure 5C; Table S12). The regulatory network of the S_I subtype may contribute to its dysregulated tumor characteristics. Such a network indicates a stronger response to cellular signals, allowing the subtype to adapt to diverse environmental stresses and therapeutic interventions. In contrast, the S_III subtype had the simplest kinase–substrate network (Figure 5E; Table S12), which may partly explain its best prognosis. Notably, CSNK2A1 exhibited significant correlations with 27 phosphosubstrates specifically within the S_I subtype (Figure 5C; Table S12), indicating it may be crucial in sustaining the malignant tumor phenotype. Our analysis identifies subtype‐specific variations in kinase–phosphosubstrate regulatory networks, suggesting the potential for personalized therapeutic strategies for CRC patients.

### 2.6 Discovery and Confirmation of Subtype‐Specific Therapeutic Agents

**FIGURE 6:**
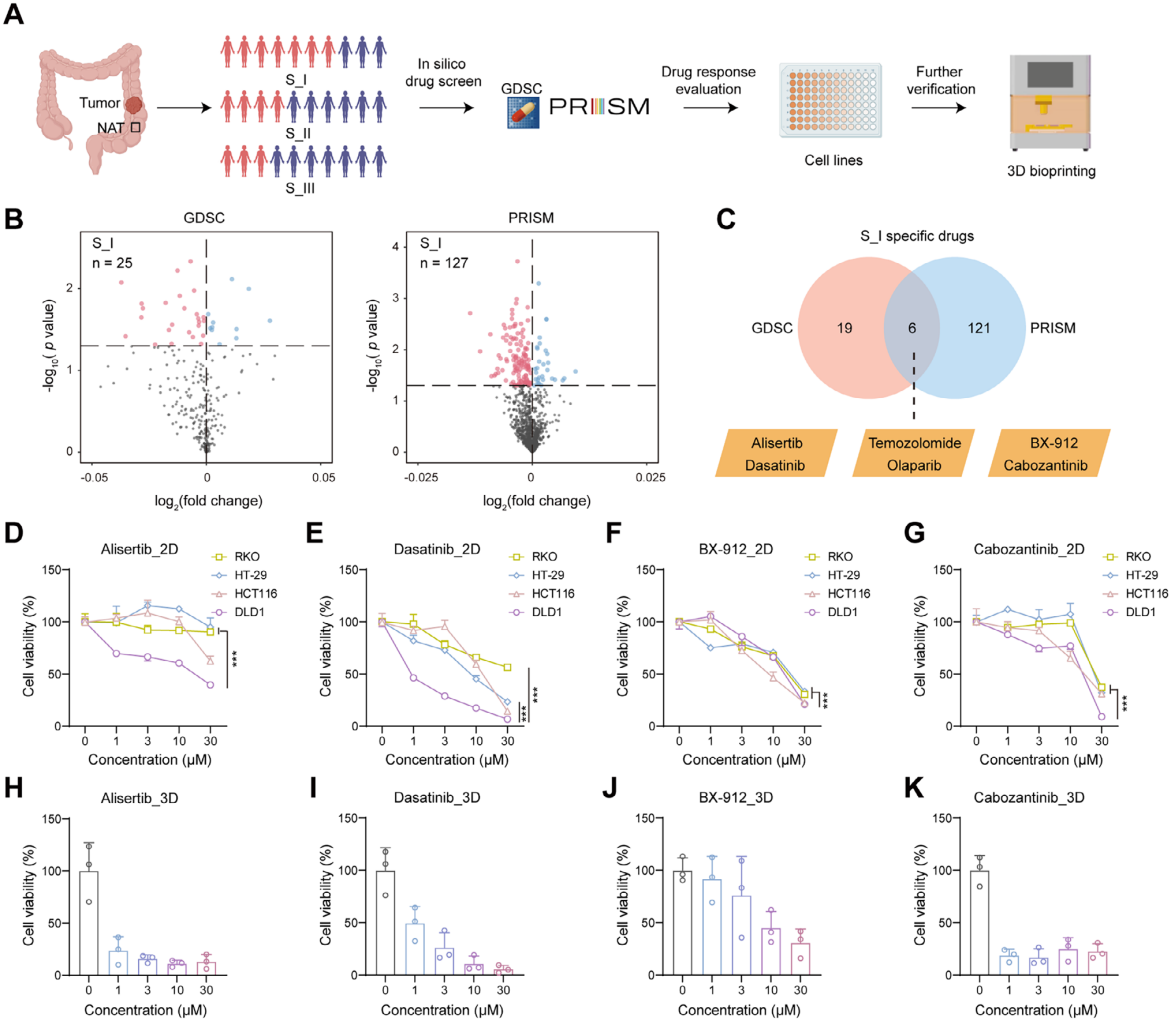
Drug prediction and validation for CRC based on defined subtype. (A) The workflow of drug prediction and validation. (B) Drugs specific to the S_I subtype were identified in the GDSC and PRISM datasets. (C) Venn diagram showing the overlap of subtype‐specific compounds for S_I. (D–G) Cell proliferation in CRC was evaluated using CCK‐8 assays after 48 h treatments with Alisertib (D), Dasatinib (E), BX‐9122 (F), and Cabozantinib (G), with three replicates per condition. Statistical significance was determined using the Student *t*‐test, with *p < 0.05, **p < 0.01, and ***p < 0.001. (H–K) Cell viability assays were conducted on patient‐derived 3D bioprinted models treated with Alisertib (H), Dasatinib (I), BX‐9122 (J), and Cabozantinib (K) for 6 days (*n* = 3 replicates).

To address the poor prognosis of the S_I subtype, we applied an integrated methodology to explore potential subtype‐specific therapeutic options (Figure 6A). Drug response data were first gathered from the Genomics of Drug Sensitivity in Cancer (GDSC) [39] and Profiling Relative Inhibition Simultaneously in Mixtures (PRISM) [40] databases. The drug sensitivity of each CRC patient was then evaluated using the calcPhenotype function from the pRRophetic package [41]. This process generated a matrix of drug sensitivities for each patient. The limma package was utilized for differential sensitivity analysis to pinpoint drugs closely linked to the S_I subtype [42]. The analysis revealed a list of potential S_I‐specific drugs, including 25 from GDSC and 127 from PRISM (Figure 6B). The selection of six candidate drugs was based on their significant association with the S_I subtype in both datasets (Figure 6C).

To enhance the identification of promising candidate drugs, validations were performed using CRC cell lines and patient‐derived three‐dimensional (3D) bioprinted models to thoroughly investigate their therapeutic potential for the S_I subtype (Figure 6D–K). Common CRC cell lines were subjected to proteomic analysis, which was used for in vitro validation experiments. The top 30 most differentially expressed proteins, sorted by log_2_(fold change), were also defined as subtype‐specific proteins at the proteomic level (Table S10). The NTP analysis based on subtype‐specific proteins revealed that the DLD‐1 and HCT116 cell lines exhibited features typical of the S_I subtype, while HT‐29 displayed characteristics associated with the S_II subtype, and RKO showed traits resembling the S_III subtype. The cell viability assay results for Alisertib, Dasatinib, BX‐912, and Cabozantinib were more consistent with our predictions (Figure 6D–G), indicating these compounds may serve as potential therapeutics for S_I patients. Besides, it was found that two drugs, Temozolomide and Olaparib, did not exhibit cytotoxicity against CRC cell lines (Figure S10A,B). Fresh tissue was collected from a CRC patient, and through RNA‐seq and NTP analysis, we determined that the patient belonged to the S_I subtype. The patient's tumor tissue was processed and then used for 3D bioprinted to assess drug sensitivity. After conducting a cell viability assay, consistent results were also observed in patient‐derived 3D bioprinted models (Figure 6H–K). Significantly, Alisertib demonstrated superior cytotoxicity against S_I subtype cells compared with S_II and S_III subtypes. Additionally, it showed exceptional efficacy in patient‐derived 3D bioprinted models.

An integrated analysis of drug sensitivity, validated in cell lines and patient‐derived 3D bioprinted models, identified Alisertib as a potential therapeutic agent for S_I subtype CRC patients. These findings suggest that personalized treatment strategies tailored to individual molecular profiles could significantly improve therapeutic outcomes and prognosis for patients with the S_I subtype of CRC. This highlights the critical need for further extensive clinical investigation and validation. The approach emphasizes the necessity of advanced models to optimize treatment and predict patient responses.

## 3 Discussion

CRC exhibits significant heterogeneity, and molecular subtyping plays a crucial role in determining prognosis and guiding personalized treatment strategies [16, 24, 43]. The lack of a molecular classification for EOCRC and LOCRC has hindered the development of effective therapeutic therapies. To address this gap, we performed an extensive multi‐omics study of CRC, integrating genomics, transcriptomics, proteomics, and phosphoproteomics. Genomics identified genetic mutations and variations in CRC, helping to identify driver genes, while transcriptomics revealed changes in gene expression, offering insights into gene regulatory networks [21]. Proteomics investigated protein alterations in CRC, exploring biomarkers and disrupted cellular functions, whereas phosphoproteomics concentrated on protein phosphorylation, revealing dysregulated signaling pathways critical for CRC progression [44]. The integration of genomics, transcriptomics, proteomics, and phosphoproteomics provided a comprehensive view of CRC, revealing complex molecular characteristics and contributing to the identification of personalized treatment strategies.

The CMS classification system provides a molecular framework for CRC [19], but it does not fully characterize the molecular heterogeneity of the CRC. This limitation primarily arises from its reliance on mRNA data alone [18]. In our study, we utilized our multi‐omics data to provide additional insights that complement the CMS classification. A total of 672 CRC samples were used to generate multi‐omics data, providing a valuable resource. Our study identified three distinct subtypes based on proteomic data, each exhibiting unique clinical prognoses and molecular characteristics. Among the identified subtypes, S_III demonstrated the longest survival, whereas S_I exhibited the shortest. Stratifying patients by survival time enhances precision in clinical management and treatment strategies [18, 45]. Significant clinicopathologic differences were observed only in age, with the S_I subtype showing a higher proportion of EOCRC. To further understand the underlying biology of these subtypes, we performed a comparative analysis of their molecular characteristics. This analysis revealed several distinctive proteins with elevated expression levels, as well as signaling pathways that were specifically enriched in each subtype. The S_I subtype, marked by a high stromal score, exhibited significant enrichment in signaling pathways related to the extracellular matrix. This is consistent with the characteristics of CMS4, which is also associated with the worst clinical outcomes [19]. Moreover, the S_I subtype showed significant enrichment of the focal adhesion signaling pathway at both the proteomic and phosphoproteomic levels. This pathway is crucial for regulating cell adhesion, migration, and invasion, processes that are known to drive cancer progression, metastasis, and treatment resistance [32, 33]. The activation of focal adhesion signaling in the S_I subtype may therefore explain the poor prognosis associated with this subtype, suggesting that targeting this pathway could offer potential clinical therapeutic benefits.

In addition to these molecular insights, we also characterized the immune landscape across the three CRC subtypes, revealing distinct tumor microenvironment profiles. The S_I subtype exhibited the highest stromal score, accompanied by overexpression of several matrix proteins and a significant increase in fibroblast infiltration. These findings suggest that the S_I subtype is characterized by ECM remodeling. Overexpression of matrix proteins such as FN1 and VCAN is commonly associated with ECM remodeling [46, 47], processes primarily regulated by activated fibroblasts [48]. Investigating the molecular mechanisms underlying fibroblast activation and ECM deposition in CRC is essential for developing more effective, targeted therapies that address the stromal components of the tumor microenvironment. In addition, the S_II subtype showed an enhanced immune response, marked by increased immune cell infiltration into the tumor microenvironment. This suggests that patients with the S_II subtype may benefit from immunotherapy, as an active immune response could improve the efficacy of immune checkpoint inhibitors and other immunomodulatory treatments [49, 50, 51]. Understanding the distinct molecular signatures, immune profiles, and survival differences among the subtypes (S_I, S_II, and S_III) can lead to more precise and effective treatment strategies. Our findings highlight the importance of molecular profiling in CRC, not only for better understanding the biology of each subtype but also for optimizing personalized therapeutic strategies to improve patient outcomes.

Protein kinases play a crucial role in regulating cellular processes such as proliferation, survival, migration, and apoptosis [52, 53]. In cancer, these kinases are often aberrantly activated, contributing to uncontrolled tumor growth and treatment resistance [52]. Consequently, protein kinases have become key targets in cancer drug therapy [54, 55]. In our study, we identified differential phosphorylation sites in each CRC subtype, suggesting their potential as subtype‐specific drug targets. Additionally, we observed that enrichments of both shared and subtype‐specific kinases, along with kinase–substrate regulatory networks, were also identified across all subtypes. We identified CSNK2A1 as a potential therapeutic target for the S_I subtype of CRC. CSNK2A1, a serine/threonine kinase, is involved in cancer‐related processes such as cell cycle regulation, apoptosis inhibition, and DNA damage repair [37, 56]. Silmitasertib, a specific inhibitor of CSNK2A1, has been shown to restore gemcitabine sensitivity in pancreatic ductal adenocarcinoma, a cancer notoriously resistant to chemotherapy [37]. Moreover, in combination with gemcitabine and cisplatin, silmitasertib has demonstrated efficacy in treating advanced and metastatic cholangiocarcinoma, another challenging cancer to treat [57]. These results indicate that targeting CSNK2A1, either independently or in combination with other treatments, may offer a novel therapeutic approach for CRC patients, particularly those with the S_I subtype. The identification of such candidate targets and inhibitors paves the way for further clinical investigation. If validated in clinical trials, these inhibitors could potentially improve treatment outcomes and prolong overall survival in CRC patients by overcoming resistance to conventional therapies and providing more targeted, effective treatment options. This highlights the importance of precision medicine and the need for more detailed molecular profiling of cancer subtypes to identify the most suitable therapeutic strategies for individual patients.

To validate therapeutic strategies for the S_I subtype of CRC, we employed CRC cell lines alongside patient‐derived 3D bioprinted models. These advanced models improve the accuracy of representing the complex interactions among cancer cells, the extracellular matrix, and stromal components, which are essential for evaluating therapeutic efficacy [58]. Our in vitro experiments demonstrated that tumors derived from the S_I subtype exhibited a significantly increased sensitivity to Alisertib compared with tumors of other molecular subtypes. Alisertib is a selective inhibitor of Aurora kinase (AURK) A, a key regulator of mitotic progression and spindle formation [24, 59]. In our study, Alisertib demonstrated advantages in both two‐dimensional (2D) cell culture systems and 3D bioprinted models. The enhanced sensitivity observed in these models suggests that AURK inhibitors, such as Alisertib, could represent an effective therapeutic option specifically for the S_I subtype of CRC. Recent studies have emphasized the importance of the immune microenvironment in cancer therapy, particularly the role of immune checkpoint proteins like PD‐L1 in suppressing antitumor immunity [60, 61]. Combining Alisertib with an anti‐PD‐L1 antibody enhances its efficacy and stimulates a stronger antitumor immune response [62]. This combination approach could be especially beneficial for the S_I subtype, as it may help overcome immune evasion mechanisms that are commonly present in CRC.

Our study provided a comprehensive multi‐omics analysis of CRC and offered valuable proteogenomic insights; several limitations should be acknowledged. First, our cohort consisted solely of resectable samples from patients who had not received any prior treatment, which limited our ability to explore the influence of the disease progression process on the stability of subtypes [21]. Future research involving longitudinal samples will be essential for assessing the stability of the subtypes. Second, our multi‐omics analysis was conducted on bulk tumor samples and their corresponding NATs, which restricted our capacity to assess cellular heterogeneity within the tumors. Incorporating single‐cell or spatial transcriptomic approaches in future studies could provide deeper insights into the complex cellular composition and spatial organization of tumors [63, 64]. Such methods would help enhance our understanding of tumor heterogeneity and its relevance to tumor biology and therapeutic strategies [65, 66]. Third, although key findings from the multi‐omics analysis were validated using cell lines and patient‐derived 3D bioprinted models, clinical trials are necessary to further validate the biological hypotheses and therapeutic predictions. Future research will incorporate real‐world clinical cases to evaluate the therapeutic efficacy of drugs for different subtypes, with the aim of refining treatment strategies and improving patient outcomes.

In conclusion, we characterized the multi‐omics landscape of EOCRC and LOCRC in Chinese CRC patients. We identified three distinct molecular subtypes, providing a foundation for personalized treatment strategies in CRC. Moreover, our comprehensive analysis represents a significant advancement in understanding the molecular alterations and underlying mechanisms of CRC tumorigenesis. The integration of extensive genomic, transcriptomic, proteomic, and phosphoproteomic data from both tumors and NATs provides a valuable resource for gaining deeper insights into the complex processes driving CRC development. Overall, our study contributes to drug discovery and the advancement of precision therapies in the field of CRC.

## 4 Material and Methods

### 4.1 Clinical Sample Acquisition

A total of 144 Chinese patients, aged 31 to 88, were recruited for the study. All the patients underwent primary resection without prior anticancer treatments. Tumors and NATs samples were obtained from patients within 30 min after resection and preserved at −80°C. Table S1 presents the baseline characteristics of the CRC patients.

### 4.2 Cell Lines

The 293T, HCT116, HT‐29, DLD‐1, and RKO cell lines were obtained from the Cell Bank of the Shanghai Institute of Cell Biology (SIBS, CAS). HCT116 and HT‐29 cells were cultured in McCoy's 5A medium (Thermo Fisher Scientific, USA), while 293T and DLD‐1 cells were cultured in DMEM medium (Meilun Biotechnology, Dalian, China). RKO cells were cultured in MEM with NEAA medium (Meilun Biotechnology). All media were supplemented with 10% fetal bovine serum (Thermo Fisher Scientific) and 1% penicillin/streptomycin (Meilun Biotechnology). Cells were incubated at 37°C with 5% CO_2_ in a humidified incubator (Thermo Fisher Scientific).

### 4.3 Proteogenomic Workflow

The proteogenomic analysis workflow of our CRC cohort is shown in Figure 1A. Table S1 provides a detailed summary of clinicopathological characteristics, including gender, age, tumor stage, and survival outcomes. Approximately 30 mg of tissue (wet‐weight) per sample was utilized for both WES and RNA‐seq. For proteomic and phosphoproteomic analyses, approximately 100 mg of tissue per sample was homogenized in a buffer containing 4% sodium dodecyl sulfate (SDS) and 100 mM Tris‐HCl at pH 7.6 [67]. The homogenized samples were stored at −80°C for further processing. In total, 162 DNA samples, 158 RNA samples, 204 protein samples, and 148 phosphorylated protein samples were used in the proteogenomic workflow (Figure 1; Figure S1A; Table S1).

### 4.4 DNA Extraction and Whole Exome Sequencing

Genomic DNA was extracted from tumors and NATs using the CTAB (hexadecyl trimethyl ammonium bromide, Sinopharm, Shanghai, China) method. DNA concentration was assessed with a Nanodrop 2000 spectrophotometer (Thermo Fisher Scientific), and its integrity was verified via 1% agarose gel electrophoresis. Libraries were prepared using the SureSelect Human All Exon V8 kit (Agilent Technologies, USA) and sequenced on the Illumina NovaSeq 6000 platform, generating 150 bp paired‐end reads. Low‐quality reads were filtered using fastp [68], and the remaining data were aligned to the GRCh37 reference genome with BWA (version 0.7.17) [69]. Somatic mutation data for the patients were shown in Table S2.

### 4.5 RNA Extraction and RNA Sequencing

RNA was extracted from fresh frozen tissues using Trizol reagent (Invitrogen, Thermo Fisher Scientific). RNA concentration, purity, and integrity were evaluated using a NanoDrop 2000 spectrophotometer and an Agilent 2100 Bioanalyzer (Agilent Technologies). The VAHTS Universal V6 RNA‐seq Kit (Vazyme, Nanjing, China) was used for library preparation, and sequencing was performed on the Illumina NovaSeq 6000 platform. Raw data underwent quality control using fastp software [68], and the resulting clean reads were aligned to the GRCh37 reference genome via HISAT2 [70]. Gene expression was measured in FPKM (fragments per kilobase of transcript per million mapped reads) [71] and subsequently log_2_‐transformed (Table S3). OE Biotech Co., Ltd. (Shanghai, China) conducted RNA‐seq and WES analyses.

### 4.6 Protein Extraction and Digestion

Tissue samples were homogenized in SDS buffer containing protease and phosphatase inhibitors (Roche, Basel, Switzerland) and sonicated on ice for 3 min with 5 s on/off cycles at 40 watts. The lysates were subjected to heating at 95°C for 4 min, followed by centrifugation at 13,000*g* for 10 min at 4°C to eliminate insoluble particles. The supernatant was further analyzed, and protein concentrations were detected by the bicinchoninic acid (BCA) assay (Thermo Fisher Scientific). For digestion, the filter‐aided sample preparation (FASP) method [67] was used. Proteins were treated with 100 mM dithiothreitol (Sigma, USA) at 56°C for 1 h and subsequently moved to 10 kDa centrifugal filter units (Millipore, USA). Samples were treated with urea buffer (8 M urea, 0.1 M Tris‐HCl, pH 8.5) and alkylated using 50 mM iodoacetamide (Sigma) for 30 min at room temperature in the dark. Proteins were digested with trypsin (Promega, USA) at a 1: 50 enzyme‐to‐protein ratio in 50 mM ammonium bicarbonate at 37°C for 18 h. Peptides were then collected by centrifugation, and their concentration was detected by the BCA assay. The peptides were dried by speed‐vacuum and desalted on MonoSpin C18 columns (GL Sciences Inc., Japan).

### 4.7 Phosphopeptides Enrichment

The phosphopeptide enrichment was performed using the High‐Select Fe‐NTA kit (Thermo Fisher Scientific) according to the manufacturer's protocol. First, peptide samples were suspended in 200 µL of binding/wash buffer and then incubated for 30 min on the equilibrated spin column. The mixtures were washed thrice with 200 µL binding/wash buffer and washed with 200 µL water. Finally, the phosphopeptides were eluted twice with 100 µL elution buffer and were dried by speed‐vacuum followed by desalting on MonoSpin C18 columns (GL Sciences Inc.). All centrifugation steps described above were carried out at 100 g at room temperature.

### 4.8 Peptides Fractionation by High‐pH Reversed‐Phase Liquid Chromatography

Peptide fractionation using high‐pH reverse‐phase liquid chromatography was employed to enhance protein and phosphopeptide identification. Peptides were examined using an Agilent 1260 system equipped with a Waters XBridge Peptide BEH C18 column (4.6 mm × 25 cm, 3.5 µm) maintained at 45°C. The mobile phases consisted of 0.2% acetonitrile in water (A) and 98% acetonitrile in water (B), both adjusted to pH 10.0 with ammonium hydroxide. The peptide separation gradient was as follows: 0–1 min, 5% B; 1–2 min, 5% B; 2–7 min, 5%–8% B; 7–42 min, 8%–18% B; 42–64 min, 18%–32% B; 64–66 min, 32%–90% B; 66–70 min, 90% B; 70–71 min, 90%–5% B; 71–87 min, 5%–5% B. Fractions were consolidated into 25 for proteomics and 15 for phosphoproteomics, then dried using a speed‐vacuum for mass spectrometry analysis.

### 4.9 Proteomic and Phosphoproteomic LC‐MS/MS Analysis

Proteomic and phosphoproteomic peptide data were obtained via mass spectrometry using the DIA method. Peptides were separated using a Thermo Fisher Scientific Easy‐nLC 1200 system equipped with a custom C18 column (75 µm × 20 cm, 3 µm) maintained at 55°C, with a flow rate of 300 nL/min. The mobile phases were composed of 0.1% formic acid in water (A) and 80% acetonitrile with 0.1% formic acid (B). Index retention time (iRT) peptides from Biognosys (Switzerland) were included in each DIA run for retention time calibration. For proteomic analysis, 1 µg of peptides was loaded and separated using a gradient of 0–57 min at 1%–25% B, 57–62 min at 25%–40% B, 62–65 min at 40%–95% B, and 65–70 min at 95% B. Mass spectrometry was operated on a Q Exactive HF‐X system (Thermo Fisher Scientific) in positive ion mode, with an MS scan range of 350–1400 m/z, a resolution of 120,000, and an AGC target of 3e6. For MS/MS, the resolution was set to 30,000, the AGC target to 1e6, and the normalized collision energy (NCE) to 28%. Fragmentation was conducted over 57 isolation windows (Table S4).

Phosphopeptides were dissolved in 10 µL of 0.1% formic acid, with 6 µL used for analysis. Phosphoproteomics samples were analyzed on the same mass spectrometer as the proteomics samples, using the following optimized gradient: 0–1 min, 1%–4% B; 1–57 min, 4%–18% B; 57–62 min, 18%–37% B; 62–68 min, 37%–45% B; 68–70 min, 45%–95% B; 70–75 min, 95%–95% B. The NCE was set to 30%, and MS parameters were identical to those used for proteomics. Fragmentation was performed across 60 isolation windows (Table S4).

### 4.10 Proteomic and Phosphoproteomic Spectral Library

A hybrid spectral library for proteomics and phosphoproteomics was generated using both data‐dependent acquisition (DDA) and DIA methods. The DDA method used the same basic parameters as the DIA method described above, with the exception that the isolation window was not applied in DDA. The comprehensive hybrid spectral library of the CRC proteome was constructed using a combination of 204 DIA runs and 75 DDA runs. Similarly, the hybrid spectral library for the CRC phosphoproteome was generated through the integration of 148 phospho‐DIA runs and 30 phospho‐DDA runs.

Spectronaut software [72] was used to generate a directDIA spectral library with the pulsar search engine from the DIA files. The analysis parameters were set as follows: enzyme/cleavage rule—trypsin/P; missed cleavages: 2; peptide length range: 2 to 52 amino acids; maximum variable modifications: 5; fixed modification—carbamidomethyl (C); variable modifications—acetyl (protein N‐term), oxidation (M). Phosphoproteomics analysis included phosphorylation at serine, threonine, and tyrosine residues as variable modifications. Phosphorylation sites with a localization probability exceeding 0.75 were selected to ensure high reliability. Nonlinear iRT calibration was used to calibrate and enable the precision iRT. The human reference FASTA file, obtained from UniProt in August 2021, was used in conjunction with the iRT fusion sequence from Biognosys. The false discovery rates (FDR) for peptides, proteins, and phosphorylation sites were maintained below 0.01, while other parameters remained at their default settings. Moreover, a DDA spectral library was analyzed using Proteome Discoverer (version 2.4.1.15, Thermo Fisher Scientific) [73] and constructed with Spectronaut (Biognosys) [72] from the DDA files. Finally, the directDIA and DDA spectral libraries were integrated for the identification of proteins, phosphoproteins, and phosphosites in CRC samples.

### 4.11 Somatic Mutation Detection and Analysis

SAMtools (version 1.9) [74] was employed to sort and index the mapped reads for further analysis. Base quality score recalibration and realignment of SNVs and INDELs were conducted using GATK (version 4.1.9.0) [75]. Somatic SNV and INDEL detection was carried out using MuTect (version 2.0) [76], and the resulting variants were annotated using ANNOVAR [77]. The mutation landscape of significantly mutated genes was visualized by the R packages maftools (version 2.16.0) [78] and ComplexHeatmap (version 3.19) [79].

### 4.12 Proteome and Phosphoproteome Data Processing

Intensities for 102 paired proteomics and 74 paired phosphoproteomics samples were obtained from Spectronaut result files (Table S5). The expression matrices for both proteomics and phosphoproteomics data were normalized by the median centering method, followed by log_2_ transformation for subsequent analysis. To guarantee enough data for imputation, the protein expression values were excluded if they had missing data in more than 50% of samples. Phosphoproteins and phosphosites were required to be expressed in at least 30% of samples. Additionally, missing values were input using the minimum values from the corresponding expression matrices.

### 4.13 Consensus Clustering for Proteomic Data

Proteomic data consensus clustering was conducted using the ConsensusClusterPlus R package (version 1.64.0) [80], with the following settings: maxK = 6, reps = 1000, pItem = 0.8, pFeature = 1, clusterAlg = “pam”, and distance = “spearman”. The median absolute deviation (MAD) was computed, and the top 15% of genes (*n* = 1035) exhibiting the highest MAD values were chosen for subtype analysis (Table S6). Using the average silhouette width and the delta plot of the cumulative distribution function (CDF) curve, the optimal number of clusters was identified as 3.

### 4.14 Somatic Copy Number Alteration Analysis

Somatic copy number variations in tumors and NATs were detected using Control‐FREEC (version 11.3) [81]. The GISTIC_2.0 algorithm (version 6.15.30) [82] was used to evaluate significant SCNAs at both arm and focal levels (Table S7). The *q*‐value threshold was set to <0.25, and a confidence level value was set to 0.9, while default parameters were employed for other settings. The individual FGA, encompassing FGL and FGG, for the CRC cohort was computed using the R package MOVICS [83]. FGA is defined as the proportion of the genome exhibiting a log_2_(copy number) greater than 0.2, compared with the entire profiled genome. *Br* denotes the count of bases in segments where |log_2_R| exceeds 0.2, while *B* signifies the total base count across all segments.

$$\begin{array}{c} R=\mathrm{Copy}\;\mathrm{number}\;\mathrm{of}\;\mathrm{segments}/2\\ \mathrm{FGA}=B_{r}/B \end{array}.$$

### 4.15 Immune Infiltration Analysis

Transcriptomic and proteomic data are essential for analyzing immune infiltration in tumors. For example, cell type deconvolution was conducted on transcriptomic data for esophagogastric junction adenocarcinoma [27], while proteomic analyses explored immune infiltration in duodenal cancer and the immune response to immunotherapy in melanoma [84, 85]. Immune cell infiltration in CRC was evaluated using the R package xCell (version 1.1.0) [86] based on both transcriptomic and proteomic data. This approach allowed for the quantification of 64 distinct immune cell signatures across CRC samples, providing an assessment of immune composition.

### 4.16 Kinase‐Substrate Enrichment Analysis

KSEA was conducted via the KSEA App (https://casecpb.shinyapps.io/ksea/) [87], utilizing phosphosite ratios between tumor and NATs. The kinase‐substrate relationships were sourced from the PhosphoSitePlus dataset [88], and *p* < 0.05 with a minimum of five substrates was applied for plot generation.

### 4.17 Drug Therapeutic Response Analysis

The study evaluated therapeutic responses using two cancer cell line drug response datasets: GDSC [39] and PRISM [40]. After excluding drugs with over 20% missing data, the GDSC dataset comprised 962 cell lines and 278 drugs, while the PRISM dataset included 480 cell lines and 1285 drugs. Expression data were obtained from the Cancer Cell Line Encyclopedia. Drug sensitivity predictions for each CRC sample were made using the pRRophetic [41] package with default settings. Differential drug response analysis was then performed across three CRC subtypes based on the predicted sensitivity data.

### 4.18 Cell Proliferation Assay

CRC cells were plated in 96‐well plates (Corning, USA) at a density of 5 × 10⁴ cells per well in 100 µL of medium and left to incubate overnight. Cells were exposed to different concentrations of Alisertib, Dasatinib, Temozolomide, Olaparib, BX‐912, and Cabozantinib (MedChemExpress, USA) for 48 h. Subsequently, 10% cell counting kit‐8 (CCK‐8, Meilun Biotechnology) reagent was added and incubated for 1 h. Absorbance was then measured at 450 nm using a Cytation 5 microplate reader (BioTek, USA). Data were normalized to the untreated control and expressed as “% Inhibition” using the formula ((OD_A_−OD_B_)/(OD_C_−OD_B_)) × 100%, where OD_A_ represents the absorbance of drug‐treated groups, OD_B_ corresponds to the blank medium group, and OD_C_ denotes the control group. IC_50_ values were calculated based on this normalization.

### 4.19 Real‐Time Quantitative PCR

Total RNA was extracted from cells using the FastPure Cell Total RNA Isolation Kit (Vazyme) following the manufacturer's instructions. The HiScript II Q RT SuperMix for qPCR Kit (Vazyme) was used to reverse transcribe the RNA into first‐strand cDNA. The resulting cDNA was then subjected to real‐time quantitative PCR using the LightCycler 96 Touch Real‐Time PCR System (Roche, USA) and ChamQ Universal SYBR qPCR Master Mix (Vazyme). GAPDH was used as the internal control. Gene expression levels were quantified relative to the control using the 2^−∆∆Ct^ method.

### 4.20 Crystal Violet Assay

HCT 116 cells (1.5 × 10^5^ cells/well) were seeded into six‐well plates (Corning) and incubated overnight. The cells were then transfected with siRNA (si‐EIF4A1) for 48 h. After discarding the supernatant, the cells were rinsed with phosphate‐buffered saline (PBS) and fixed with a 4% paraformaldehyde solution for 30 min. Following fixation, the cells were stained with crystal violet for 15 min. Images were captured using the Cytation 5 microplate reader system (BioTek), and the absorbance was measured at 595 nm after dissolving the cells in a 10% acetic acid solution.

### 4.21 Transwell Migration Assays

HCT 116 cells (8 × 10^4^ cells/well) were resuspended in 200 µL of serum‐free medium and placed in the upper chamber of a transwell system (Corning). The lower chamber was filled with 600 µL of complete medium supplemented with 20% FBS. After 48 h of incubation, the cells were rinsed with PBS and fixed with a 4% paraformaldehyde solution for 30 min. Following fixation, the cells were stained with crystal violet for 15 min. The cells on the upper surface of the membrane were gently wiped away using cotton swabs. Images were captured using the Cytation 5 microplate reader system (BioTek).

### 4.22 Patient‐Derived 3D Bioprinted Model

Tumor tissues were collected from CRC patients during surgical resection. The tissues were cut into 1–3 mm fragments and rinsed with Dulbecco's phosphate‐buffered saline (DPBS, Gibco, USA). The tissues were enzymatically dissociated into a single‐cell suspension using collagenase type I (Yeasen, Shanghai, China) at 37°C for 1 h, followed by the addition of a medium containing fetal bovine serum to halt the reaction. In 3D bioprinting, bioinks were formulated by combining 8% gelatin methacrylate with 0.2% lithium phenyl‐2,4,6‐trimethylbenzoylphosphonate for light‐curing purposes. The cell suspension and bioinks were mixed in equal parts and printed into a 96‐well plate (Corning) using a digital light processing (DLP)‐based 3D bioprinter (Cyberiad Biotechnology, Shanghai, China) for crosslinking. After confirming construct viability, drug sensitivity testing was performed with Alisertib, Dasatinib, Temozolomide, Olaparib, BX‐912, and Cabozantinib for 6 days. The CellTiter‐Glo 3D assay (Promega) was utilized to evaluate cell viability.

### 4.23 Quantification and Statistical Analysis

Statistical analyses of multi‐omics data employed tests such as the Wilcoxon rank‐sum test, Student's *t*‐test, Kruskal–Wallis test, chi‐square test, and Fisher's exact test. Spearman's correlation evaluated data relationships, and overall survival was estimated using the Kaplan–Meier method with the log‐rank test. In box‐and‐whisker plots, the box indicates the interquartile range, and the median is shown as a line. Functional experiments were conducted a minimum of three times, with results expressed as mean ± standard deviation (SD). Statistical analyses were conducted using GraphPad Prism 8.0, considering *p* < 0.05 as the threshold for statistical significance.

## Author Contributions

Xin Guo, Xin Luan, Haiyang Zhou, and Weidong Zhang collaboratively planned and designed the study proposal. Xin Guo, Hongwei Zhang, Yan Jin, Jiayi Lin, and Min Tang performed experiments. Xin Guo and Saisai Tian performed data bioinformatics analysis. Xin Guo, Xinxing Li, Jiayi Lin, Sanhong Liu, Xin Luan, Haiyang Zhou, and Weidong Zhang analyzed and discussed data. Xin Guo, Xinxing Li, Anqi Wang, Ce Bian, and Haiyang Zhou collected and classified clinical samples. Xin Guo, Saisai Tian, Min Tang, Lijun Zhang, Xin Luan, Haiyang Zhou, and Weidong Zhang collaboratively authored and edited the manuscript. All authors have seen and approved the contents of the manuscript.

## Ethics Statement

This study was approved by the Ethics Committee (2022SL062) of Changzheng Hospital (Shanghai, China), and informed consent was obtained from all patients.

## Conflicts of Interest

The authors declare no conflicts of interest.

## Supporting information




**Figure S1**: Quality assessments of genomic, transcriptomic, proteomic, and phosphoproteomic data. (A) Venn diagram summary of the CRC patients in WES, RNA‐seq, proteomic, and phosphoproteomic analysis. A total of 102 pairs of tumors and NATs were used for proteome profiling (pink circle). A total of 74 pairs of tumors and NATs were used for phosphoproteome profiling (green circle). A total of 81 pairs of CCAs and NATs were performed on WES (purple circle). A total of 79 pairs of CCAs and NATs were used for RNA‐seq analysis (blue circle). (B) Density plot showing the distribution of RNA abundances in tumors and NATs. A unimodal distribution is observed. All of the samples pass quality control. (C, D) The Venn diagram showing the identification of RNA (C) and proteins (D) in tumors and NATs, and the overlap of their shared identified proteins. (E, F) Overview of the phosphoproteomic profile of CRC patients. The Venn diagram showing the identification of phosphoproteins (E) and phosphosites (F) in tumors and NATs, and their shared identification numbers. (G) The average Spearman's correlation coefficients of 293T samples on the proteome platform were 0.96, exhibiting good reproducibility between these repeat experiments. (H) The average Spearman's correlation coefficients of 293T samples on the phosphoproteome platform were 0.93, exhibiting good reproducibility between these repeat experiments.
**Figure S2**: Profiles of SCNAs in CRC. (A) All detected somatic copy number alterations in CRC. Genes are ordered by chromosomal location (*y*‐axis). (B, C) Copy number amplifications (B) and deletions (C) analysis. The panel depicts genomic positions of amplified and deleted regions, with x‐axes representing the normalized amplification and deletion signals (top) and significance by *q*‐value (bottom). The green lines represent the significance cutoff at *q*‐value = 0.25.
**Figure S3**: The multivariate statistical analysis of CRC at multi‐omics levels. (A, B) Principal‐component analysis (PCA, A) and partial least squares discrimination analysis (PLS‐DA, B) plots showing RNA abundance in CRC tumors (red) and NATs (blue). (C, D) PCA (C) and PLS‐DA (D) plots showing proteomic expression in tumors (red) and NATs (blue). (E, F) PCA (E) and PLS‐DA (F) plots showing phosphoprotein abundance in tumors (red) and NATs (blue).
**Figure S4**: Prognosis and molecular characteristics of EOCRC and LOCRC. (A, B) Kaplan–Meier survival curves for overall survival in EOCRC and LOCRC are presented for our cohort (A) and the CPTAC cohort (B), including log‐rank test *p*‐values. (C) The differentially expressed proteins between EOCRC and LOCRC, with columns representing patient samples and rows representing proteins. (D) Functional enrichment analysis of upregulated (light red) and downregulated (light blue) proteins in EOCRC compared with LOCRC.
**Figure S5**: Consensus clustering of proteomic profiles in the CRC cohort. (A–E) Identification of clusters based on proteomic data of the CRC cohort (*n* = 102) using the ConsensusClusterPlus R package based on their abundance. *K* was set from 2 to 6, and the consensus matrices are displayed. (F–H) Consensus cumulative distribution function (CDF) plot (F), as well as delta area (change in CDF area) plot (G), and tracking plot are shown (H). (I) Multivariate analyses (Cox proportional hazards models) of subtypes and clinicopathological characteristics (gender, age, TNM staging, MSI status).
**Figure S6**: Immune infiltration analysis across three subtypes. (A, B) Comparisons of stroma score (A) and immune score (B) in the S_I, S_II, and S_III subtypes. (C) Uniform manifold approximation and projection (UMAP) representation of the dataset GSE132465, and the stroma score (D) and immune score (E) in the three subtypes. (F) Enrichment analysis of signal pathways of different subtypes in the dataset GSE132465.
**Figure S7**: Immunohistochemical analysis of (A) α‐SMA (a marker of fibroblasts), (B) CD68 (macrophages), (C) CD4 (T helper cells), (D) CD8 (cytotoxic T cells), and (E) CD19 (B cells) across three subtypes (S_I, S_II & S_III).
**Figure S8**: (A) Workflow for identifying signature proteins in the S_I subtype significantly linked to patient survival. (B) The hazard ratio for each signature protein in multivariate Cox regression analysis. Crystal violet staining (C) and transwell migration (F) assays of HCT116 cells after EIF4A1 knockdown. Statistical analysis of relative mRNA expression (D), cell viability (E), and cell migration (G) of HCT116 cells after EIF4A1 knockdown (mean ± SD, *n* = 3, ***p < 0.001).
**Figure S9**: Classification prediction of unknown RNA samples and evaluation of kinase activity in the CRC cohort. (A) The transcriptome data were used to make subtyping predictions of unknown samples (S_I, *n* = 16; S_II, *n* = 14; S_III, *n* = 12). (B–D) Evaluation of kinase activities by KSEA across the S_I (B), S_II (C), and S_III (D) proteomic subtypes.
**Figure S10**: Drug validation for CRC based on defined subtype. (A, B) CRC cell proliferation was detected using CCK‐8 assays after treatment with Temozolomide (A) and Olaparib (B) for 48 h (*n* = 3 replicates).
**Table S4**: The DIA isolation windows for MS runs.

**Table S1**: Clinicopathologic information in the CRC cohort.

**Table S2**: Somatic mutations information in the CRC cohort.

**Table S3**: Transcriptomic data in the CRC cohort.

**Table S5**: Proteomic and phosphoproteomic data in the CRC cohort.

**Table S6**: Different proteomic subtypes of CRC and their associations with clinical prognosis.

**Table S7**: Genomic alterations in the CRC cohort.

**Table S8**: The 293T cell samples as quality controls for the mass spectrometry in proteomic and phosphoproteomic analyses.

**Table S9**: Immune infiltration across different proteomic subtypes.

**Table S10**: Subtype‐specific genes among the three proteomic subtypes.

**Table S11**: Comparison of gene mutation among the three subtypes.

**Table S12**: A comparative analysis of the phosphoproteomic characteristics across the three proteomic subtypes.

## Data Availability

The raw data from WES and RNA‐seq have been deposited in the Genome Sequence Archive (GSA) database under accession number HRA013067 (https://ngdc.cncb.ac.cn/gsa‐human/), which is part of the National Genomics Data Center, China National Center for Bioinformation/Beijing Institute of Genomics, Chinese Academy of Sciences. The proteomic and phosphoproteomic data have been deposited in the iProX database, National Center for Protein Sciences/Beijing Protein Research Center (ProteomeXchange ID: PXD067508, http://www.iprox.org). All other data are available in the main text or the Supporting Information Materials. Any additional information required to reanalyze the data reported in this paper is available from the corresponding author upon reasonable request.
