# Supplementary material for: Integrated Multi‐Omics Profiling to Characterize Molecular Subtypes and Reveal Potential Therapeutic Strategies for Colorectal Cancer
Source: MedComm (2020). 2025 Dec 8;6(12):e70492. doi: 10.1002/mco2.70492 (PMC12686132; doi:10.1002/mco2.70492)
Supplement: Supplementary file 1 — Figure S1: Quality assessments of genomic, transcriptomic, proteomic, and phosphoproteomic data. (A) Venn diagram summary of the CRC patients in WES, RNA‐seq, proteomic, and phosphoproteomic analysis. A total of 102 pairs of tumors and NATs were used for proteome profiling (pink circle). A total of 74 pairs of tumors and NATs were used for phosphoproteome profiling (green circle). A total of 81 pairs of CCAs and NATs were performed on WES (purple circle). A total of 79 pairs of CCAs and NATs were used for RNA‐seq analysis (blue circle). (B) Density plot showing the distribution of RNA abundances in tumors and NATs. A unimodal distribution is observed. All of the samples pass quality control. (C, D) The Venn diagram showing the identification of RNA (C) and proteins (D) in tumors and NATs, and the overlap of their shared identified proteins. (E, F) Overview of the phosphoproteomic profile of CRC patients. The Venn diagram showing the identification of phosphoproteins (E) and phosphosites (F) in tumors and NATs, and their shared identification numbers. (G) The average Spearman's correlation coefficients of 293T samples on the proteome platform were 0.96, exhibiting good reproducibility between these repeat experiments. (H) The average Spearman's correlation coefficients of 293T samples on the phosphoproteome platform were 0.93, exhibiting good reproducibility between these repeat experiments. Figure S2: Profiles of SCNAs in CRC. (A) All detected somatic copy number alterations in CRC. Genes are ordered by chromosomal location (y‐axis). (B, C) Copy number amplifications (B) and deletions (C) analysis. The panel depicts genomic positions of amplified and deleted regions, with x‐axes representing the normalized amplification and deletion signals (top) and significance by q‐value (bottom). The green lines represent the significance cutoff at q‐value = 0.25. Figure S3: The multivariate statistical analysis of CRC at multi‐omics levels. (A, B) Principal‐component analysis ( [file MCO2-6-e70492-s012.docx]

**Supplementary Information**

**Integrated multi-omics profiling to characterize molecular subtypes and reveal potential therapeutic strategies for colorectal cancer**

Xin Guo^1,4,#^, Saisai Tian^3,#^, Xinxing Li^6,#^, Hongwei Zhang^1^, Anqi Wang^2^, Yan Jin^1^, Ce Bian^2^, Jiayi Lin^1^, Sanhong Liu^1^, Min Tang^1^, Lijun Zhang^1^, Xin Luan^1,^*, Haiyang Zhou^1,2,^*, and Weidong Zhang^1,3,5,^*

^1^Shanghai Frontiers Science Center of Chinese Medicine Chemical Biology, Institute of Interdisciplinary Integrative Medicine Research and Shuguang Hospital, Shanghai University of Traditional Chinese Medicine, Shanghai 201203, China

^2^Division of Colorectal Surgery, Changzheng Hospital, Naval Medical University, Shanghai 200433, China

^3^School of Pharmacy, Naval Medical University, Shanghai 200433, China

^4^School of Pharmacy, Shanghai Jiao Tong University, Shanghai 200240, China

^5^State Key Laboratory for Quality Ensurance and Sustainable Use of Dao-di Herbs, Institute of Medicinal Plant Development, Chinese Academy of Medical Science and Peking Union Medical College, Beijing 100193, China

^6^Department of Gastrointestinal Surgery, Tongji Hospital, Tongji University School of Medicine, Shanghai 200065, China

^#^Xin Guo, Saisai Tian, and Xinxing Li contributed equally to this work.

*Corresponding autho­­rs: Xin Luan (luanxin@shutcm.edu.cn), Haiyang Zhou (haiyang1985_1@aliyun.com), and Weidong Zhang ([wdzhangy@hotmail.com](mailto:wdzhangy@hotmail.com))

**
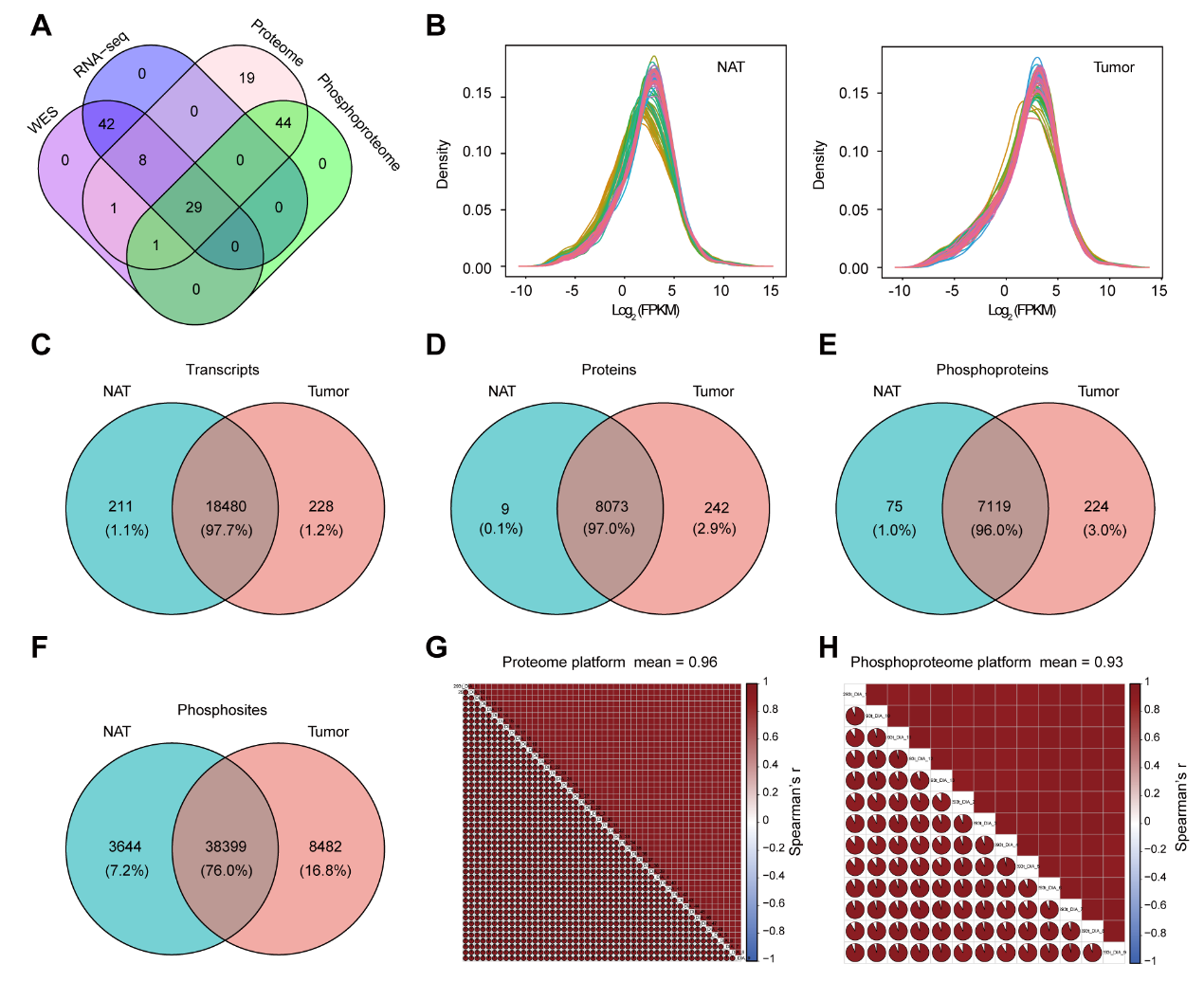
**

**Figure S1** Quality assessm­­ents of genomic, transcriptomic, proteomic, and phosphoproteomic data. (A) Venn diagram summary of the CRC patients in WES, RNA-seq, proteomic, and phosphoproteomic analysis. A total of 102 pairs of tumors and NATs were used for proteome profiling (pink circle). A total of 74 pairs of tumors and NATs were used for phosphoproteome profiling (green circle). A total of 81 pairs of CCAs and NATs were performed WES (purple circle). A total of 79 pairs of CCAs and NATs were used for RNA-seq analysis (blue circle). (B) Density plot showing the distribution of RNA abundances in tumors and NATs. A unimodal distribution is observed. All of the samples pass quality control. (C-D) The Venn diagram showing the identification of RNA (C) and proteins (D) in tumors and NATs, and the overlap of their shared identified proteins. (E-F) Overview of the phosphoproteomic profile of CRC patients. The Venn diagram showing the identification of phosphoproteins (E) and phosphosites (F) in tumors and NATs, and their shared identification numbers. (G) The average Spearman’s correlation coefficients of 293T samples on the proteome platform were 0.96, exhibiting good reproducibility between these repeat experiments. (H) The average Spearman’s correlation coefficients of 293T samples on the phosphoproteome platform were 0.93, exhibiting good reproducibility between these repeat experiments.


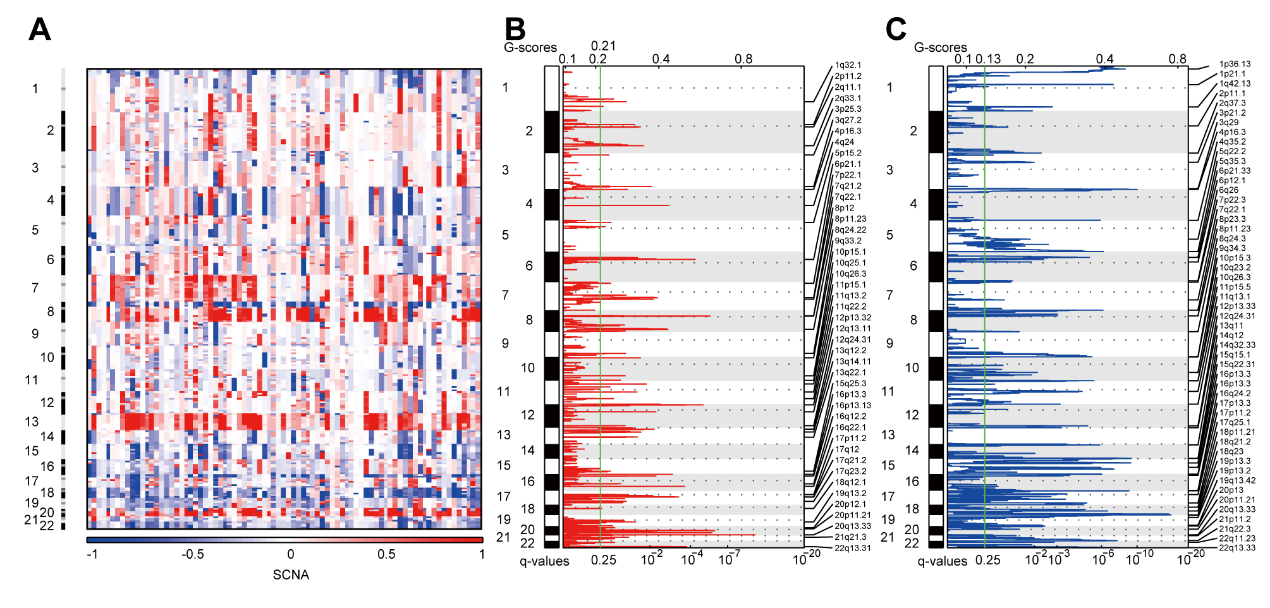


**Figure S2** Profiles of SCNAs in CRC. (A) All detected somatic copy number alterations in CRC. Genes are ordered by chromosomal location (y axis). (B-C) Copy number amplifications (B) and deletions (C) analysis. The panel depicts genomic positions of amplified and deleted regions, with x-axes representing the normalized amplification and deletion signals (top) and significance by q-value (bottom). The green lines represent the significance cutoff at q-value = 0.25.


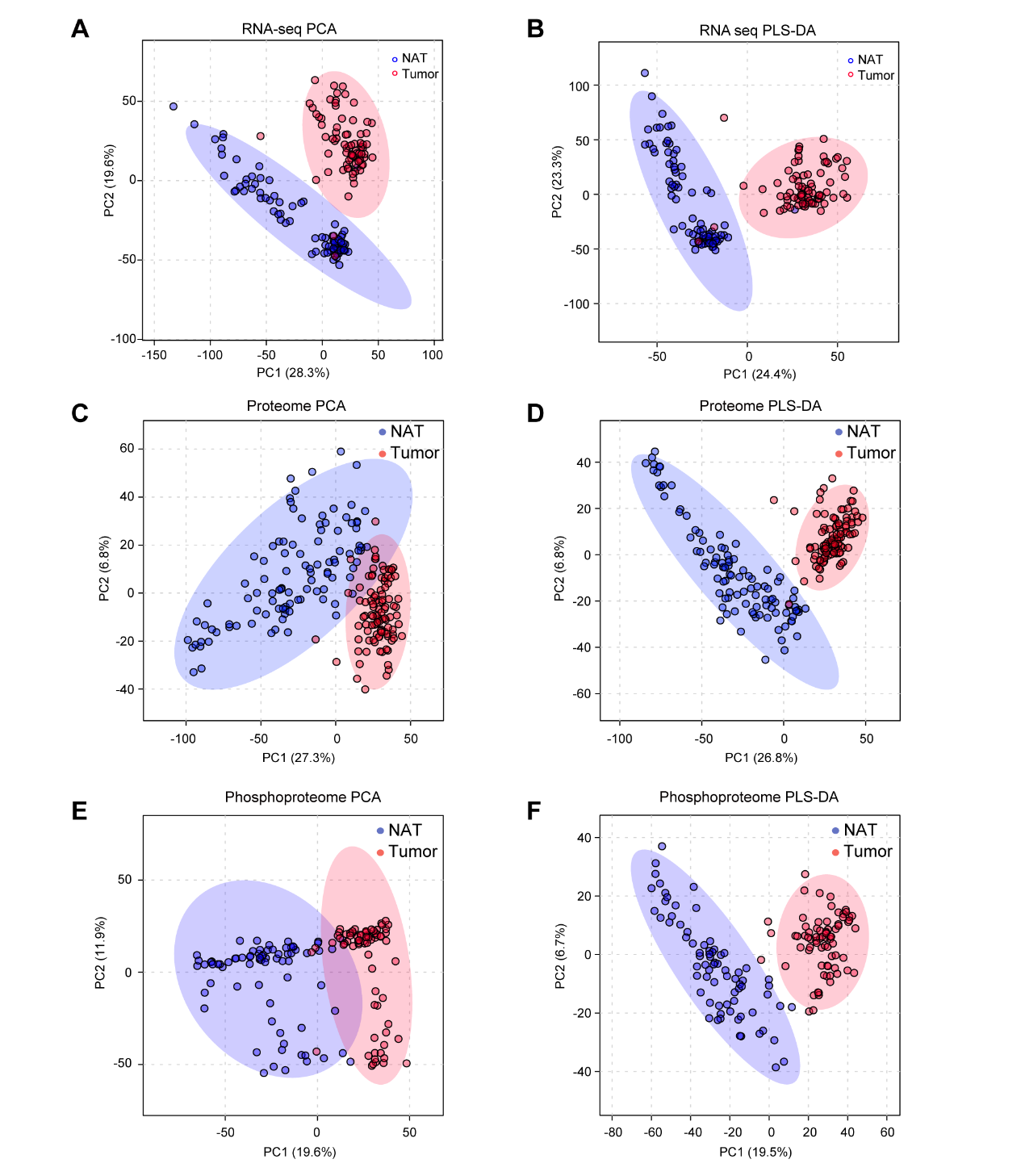


**Figure S3** The multivariate statistical analysis of CRC at multi-omics levels. (A-B) Principal-component analysis (PCA, A) and partial least squares discrimination analysis (PLS-DA, B) plots showing RNA abundance in CRC tumors (red) and NATs (blue). (C-D) PCA (C) and PLS-DA (D) plots showing proteomic expression in tumors (red) and NATs (blue). (E-F) PCA (E) and PLS-DA (F) plots showing phosphoprotein abundance in tumors (red) and NATs (blue).


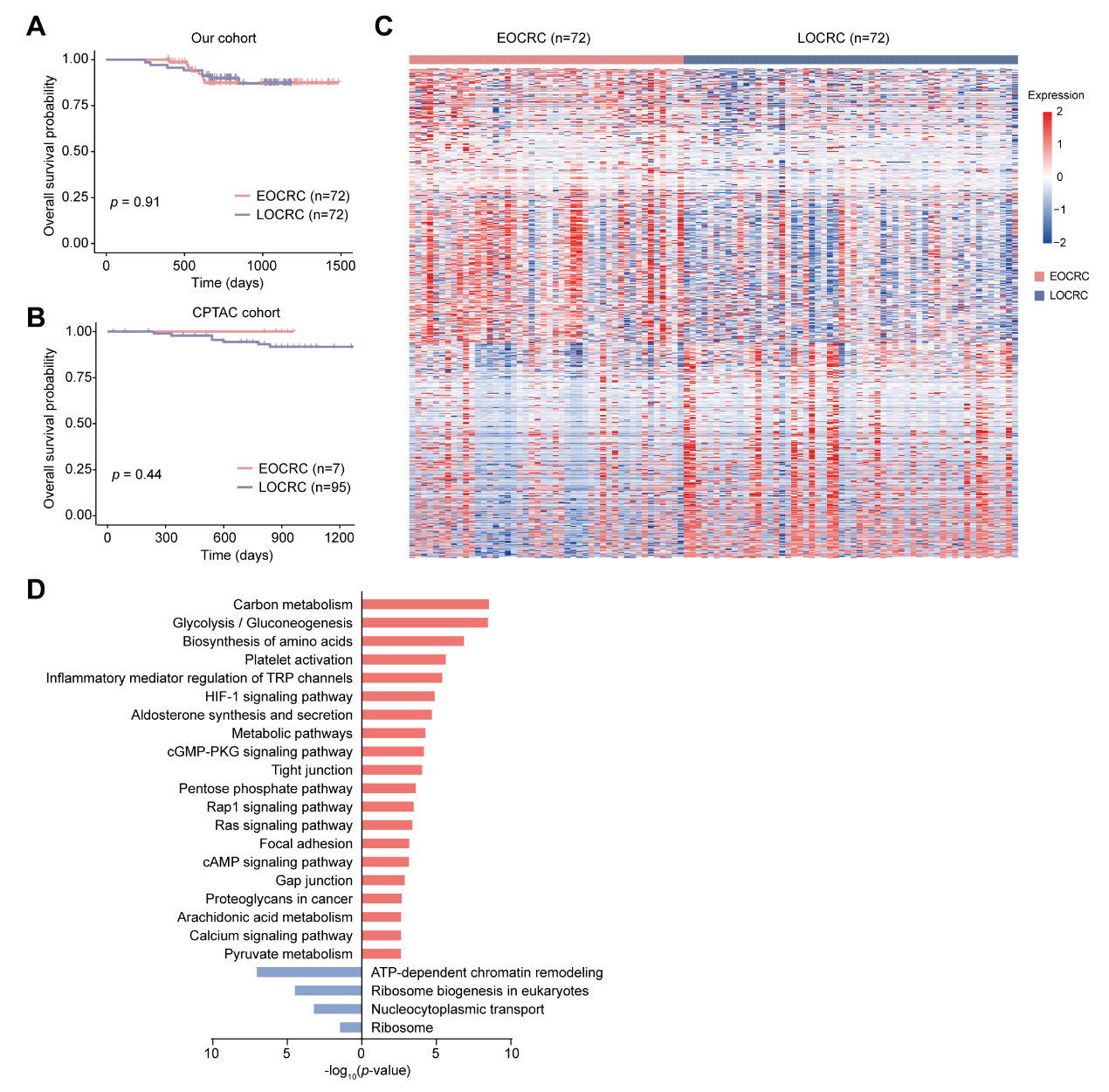


**Figure S4** Prognosis and molecular characteristics of EOCRC and LOCRC. (A-B) Kaplan-Meier survival curves for overall survival in EOCRC and LOCRC are presented for our cohort (A), and CPTAC cohort (B), including log-rank test *p*-values. (C) The differentially expressed proteins between EOCRC and LOCRC, with columns representing patient samples and rows representing proteins. (D) Functional enrichment analysis of upregulated (light red) and downregulated (light blue) proteins in EOCRC compared to LOCRC.


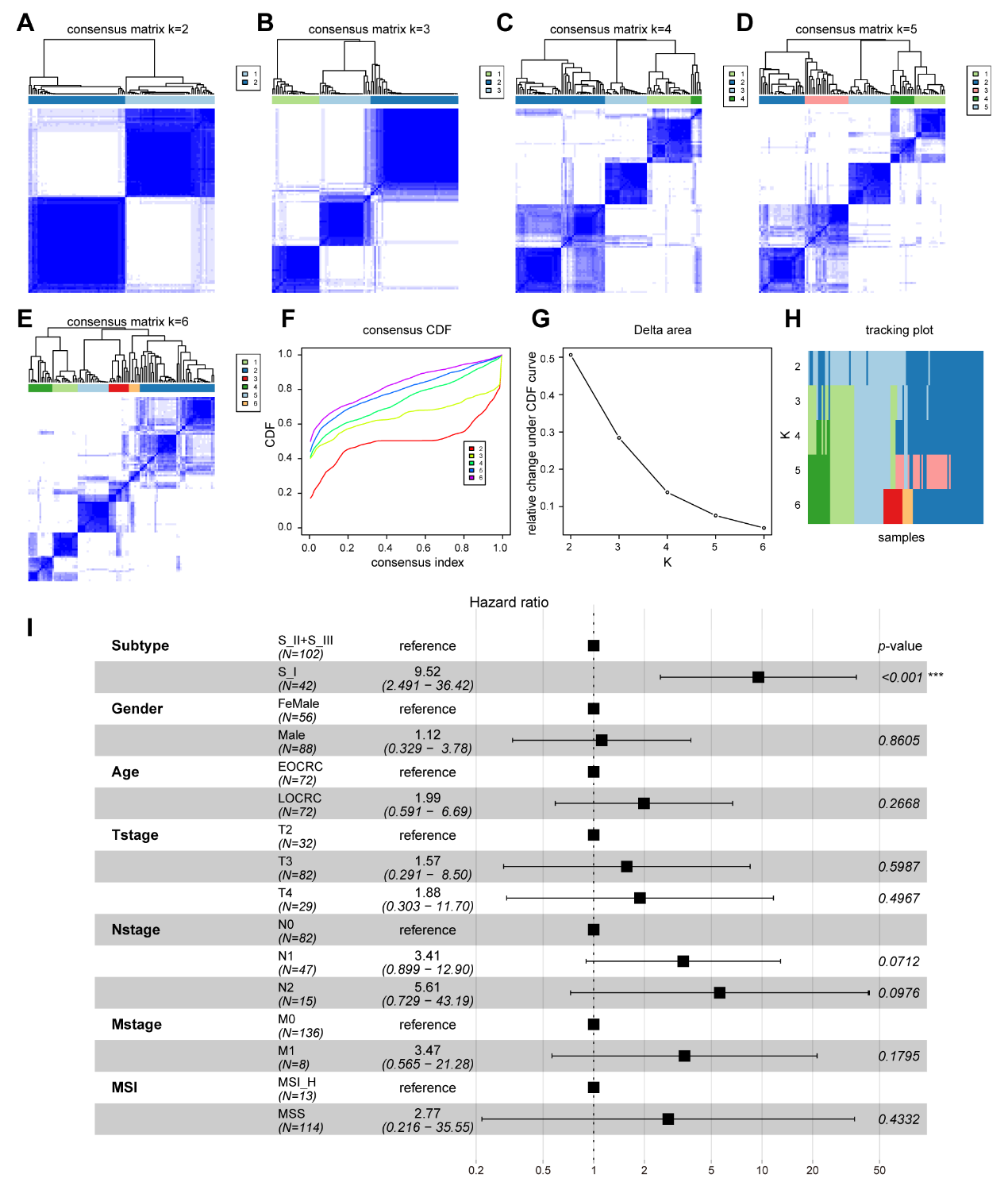


**Figure S5** Consensus clustering of proteomic profiles in the CRC cohort. (A-E) Identification of clusters based on proteomic data of the CRC cohort (n = 102) using ConsensusClusterPlus R package upon their abundance. K was set from 2 to 6, and the consensus matrices are displayed. (F-H) Consensus cumulative distribution function (CDF) plot (F), as well as delta area (change in CDF area) plot (G), and tracking plot are shown (H). (I) Multivariate analyses (Cox proportional hazards models) of subtypes and clinicopathological characteristics (gender, age, TNM staging, MSI status).

­­


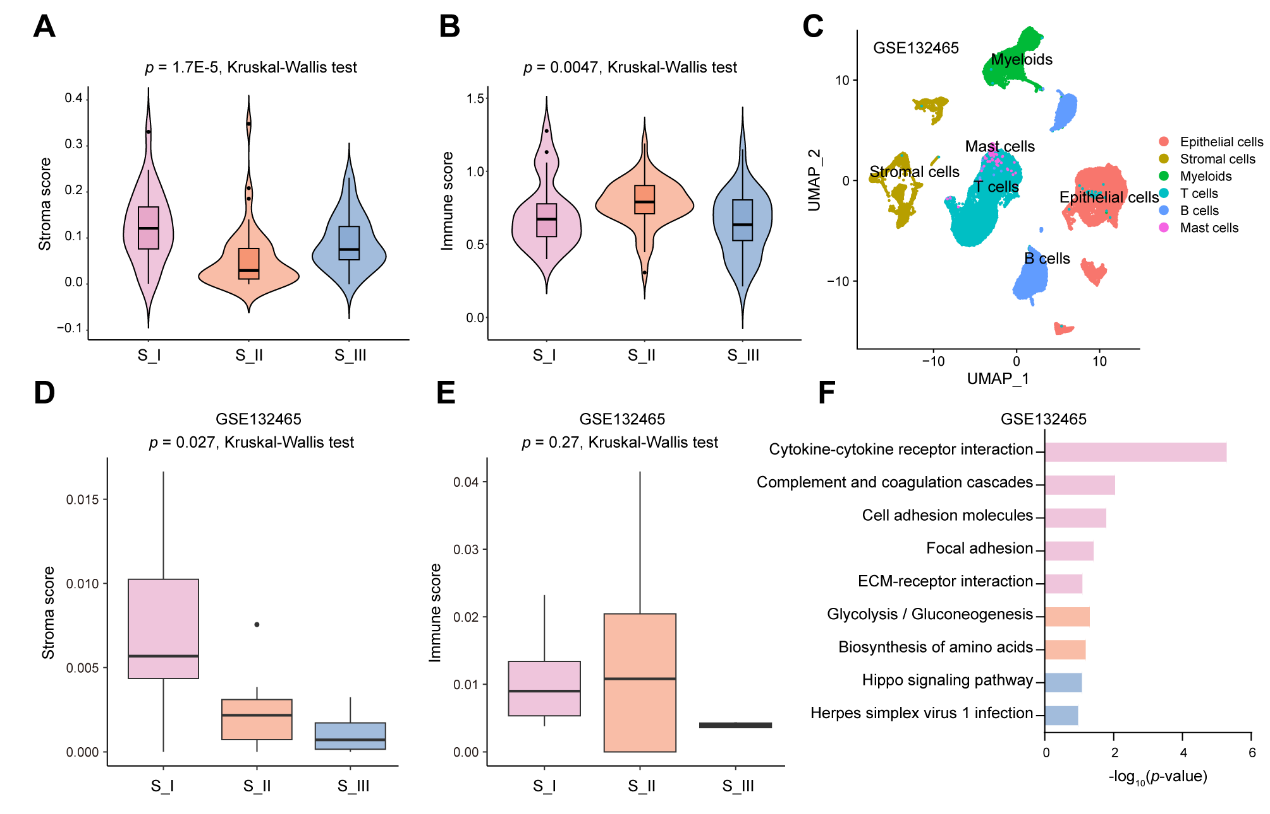


**Figure S6** Immune infiltration analysis across three subtypes. (A-B) Comparisons of stroma score (A) and immune score (B) in the S_I, S_II, and S_III subtypes. (C) Uniform manifold approximation and projection (UMAP) representation of the dataset GSE132465, and the stroma score (D) and immune score (E) in the three subtypes. (F) Enrichment analysis of signal pathways of different subtypes in the dataset GSE132465.


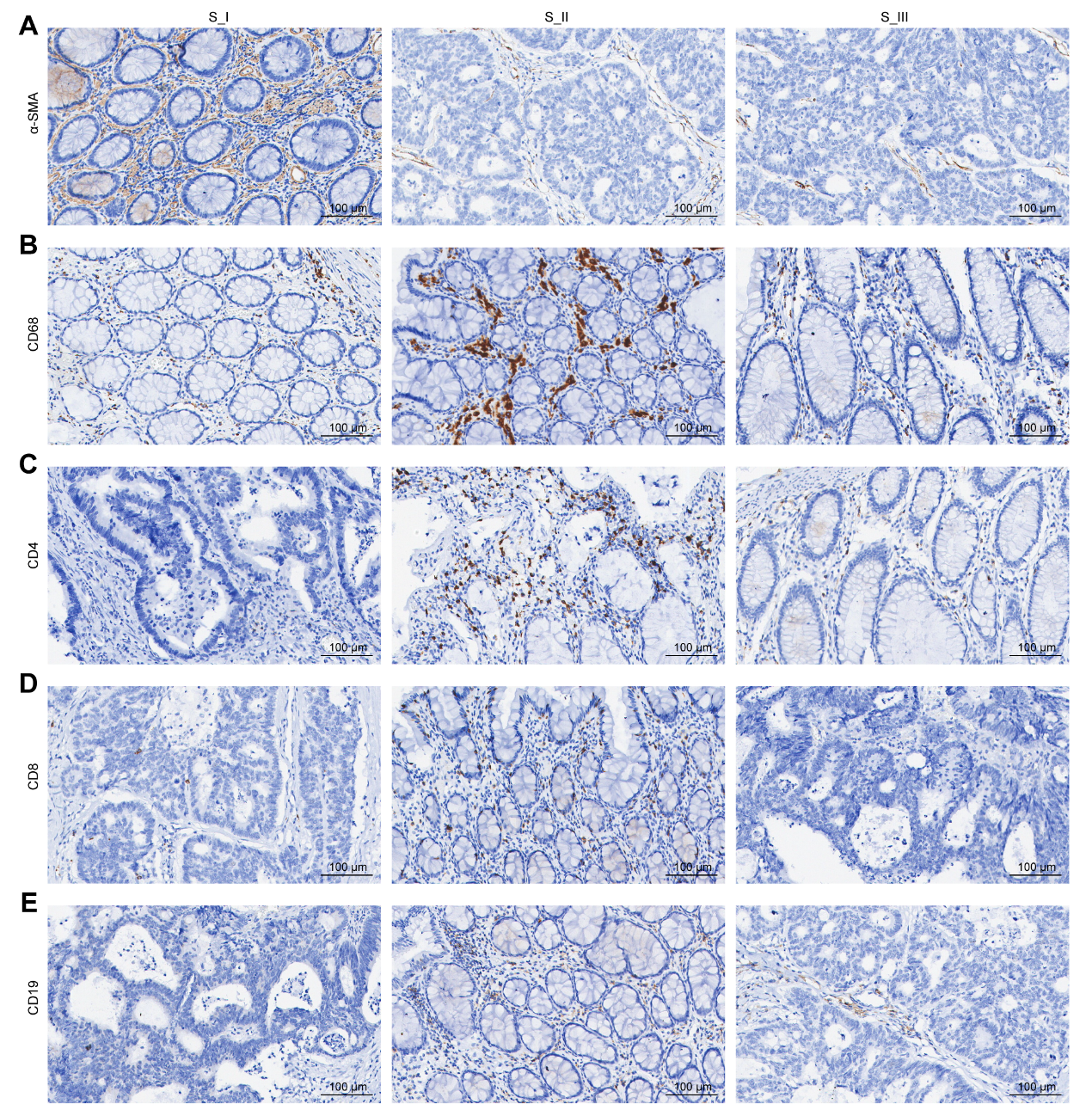


**Figure S7** Immunohistochemical analysis of (A) α-SMA (a marker of fibroblasts), (B) CD68 (macrophages), (C) CD4 (T helper cells), (D) CD8 (cytotoxic T cells), and (E) CD19 (B cells) across three subtypes (S_I, S_II & S_III).


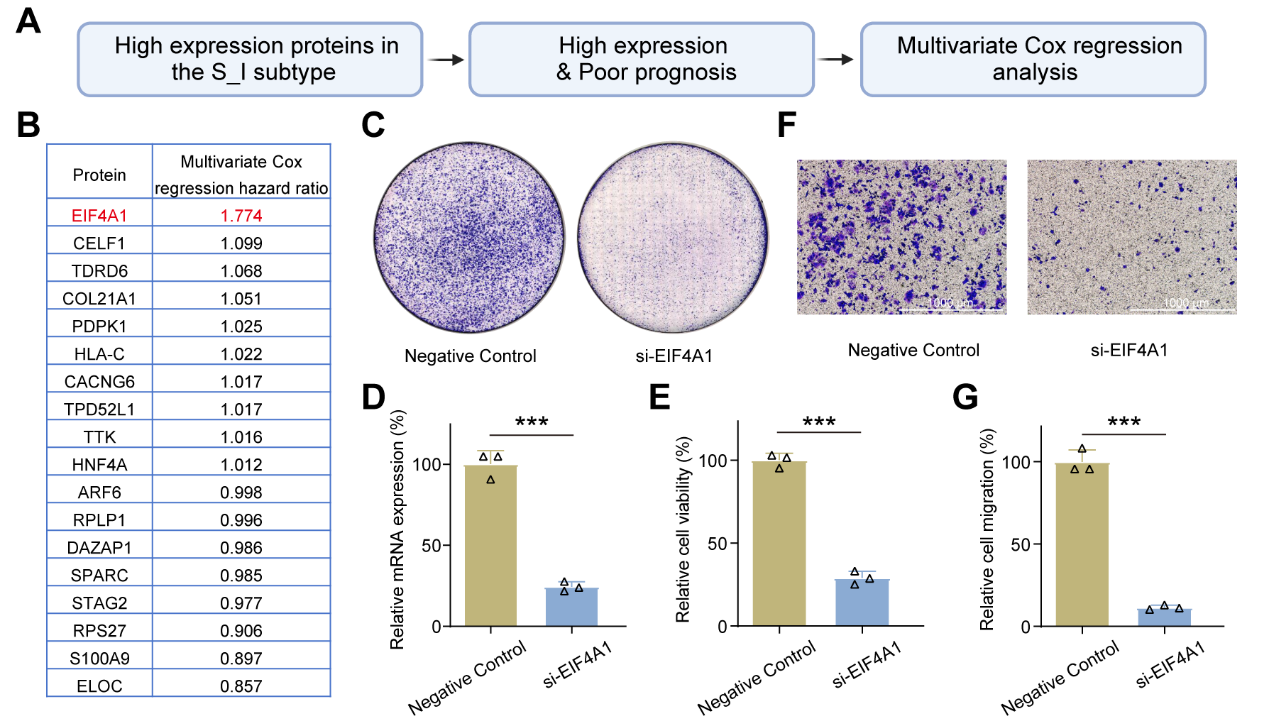


**Figure S8** (A) Workflow for identifying signature proteins in the S_I subtype significantly linked to patient survival. (B) The hazard ratio for each signature protein in multivariate Cox regression analysis. Crystal violet staining (C) and transwell migration (F) assays of HCT116 cells after EIF4A1 knockdown. Statistical analysis of relative mRNA expression (D), cell viability (E), and cell migration (G) of HCT116 cells after EIF4A1 knockdown (mean ± SD, n=3, *** *p* < 0.001).


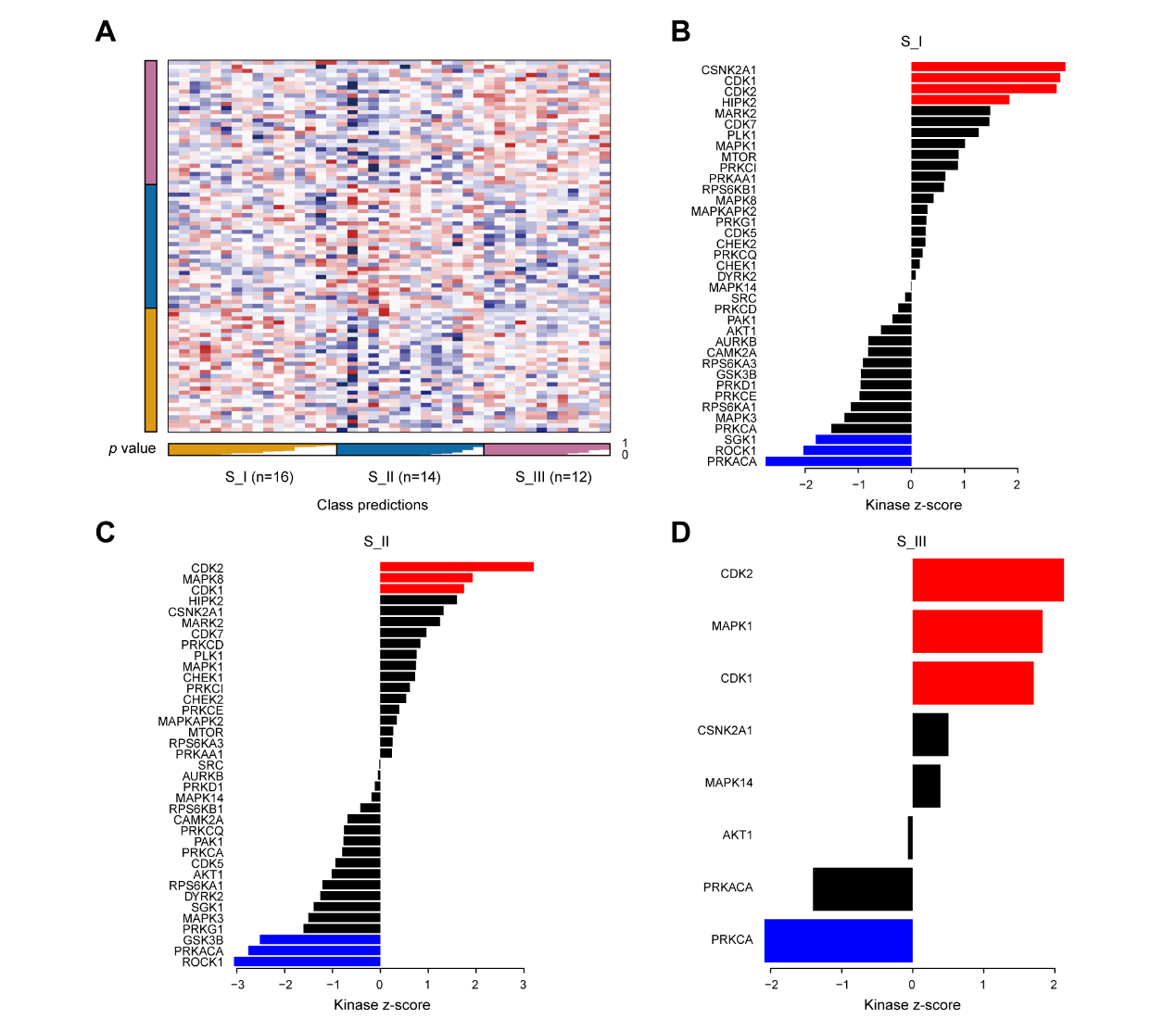


**Figure S9** Classification prediction of unknown RNA samples and evaluation of kinase activity in the CRC cohort. (A) The transcriptome data were used to make subtyping prediction of unknown samples (S_I, n = 16; S_II, n = 14; S_III, n = 12). (B-D) Evaluation of kinase activities by KSEA across the S_I (B), S_II (C), and S_III (D) proteomic subtypes.


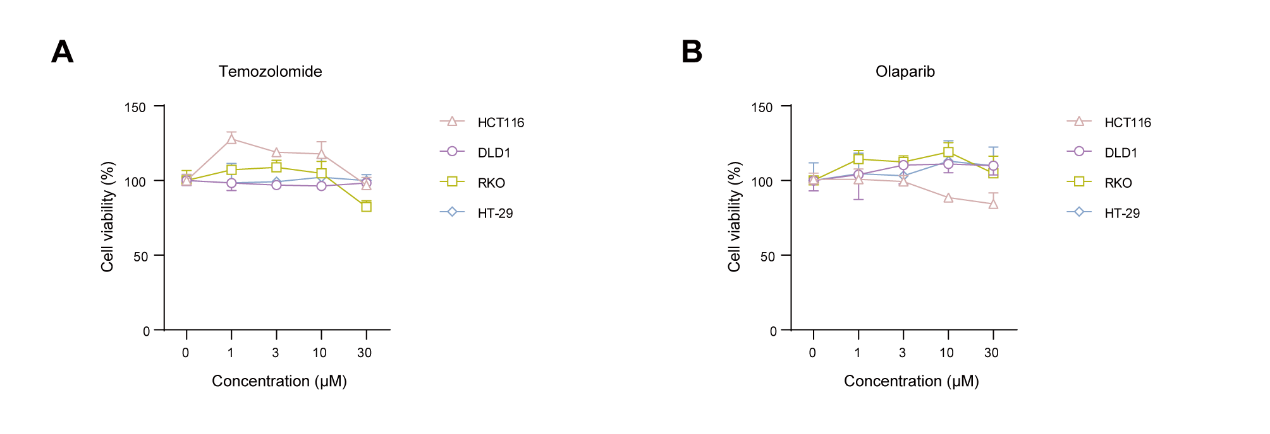


**Figure S10** Drug validation for CRC based on defined subtype. (A-B) CRC cell proliferation were detected using CCK-8 assays after treatment with Temozolomide (A) and Olaparib (B) for 48 h (n = 3 replicates).

**Table S4.** The DIA isolation windows for MS runs.

| No. | isolation window _ proteome (n = 57) | central m/z _ proteome | No. | isolation window _ phosphoproteome (n = 60) | central m/z _ phosphoproteome |
| --- | --- | --- | --- | --- | --- |
|  | Full-MS 350-1400 | / |  | Full-MS 350-1400 | / |
| 1 | 59 | 379.5 | 1 | 60 | 380 |
| 2 | 27 | 422.5 | 2 | 28 | 424 |
| 3 | 19 | 445.5 | 3 | 18 | 447 |
| 4 | 19 | 464.5 | 4 | 18 | 465 |
| 5 | 13 | 480.5 | 5 | 13 | 480.5 |
| 6 | 15 | 494.5 | 6 | 15 | 494.5 |
| 7 | 11 | 507.5 | 7 | 11 | 507.5 |
| 8 | 14 | 520 | 8 | 14 | 520 |
| 9 | 12 | 533 | 9 | 12 | 533 |
| 10 | 13 | 545.5 | 10 | 13 | 545.5 |
| 11 | 12 | 558 | 11 | 12 | 558 |
| 12 | 11 | 569.5 | 12 | 11 | 569.5 |
| 13 | 10 | 580 | 13 | 10 | 580 |
| 14 | 12 | 591 | 14 | 12 | 591 |
| 15 | 11 | 602.5 | 15 | 11 | 602.5 |
| 16 | 12 | 614 | 16 | 12 | 614 |
| 17 | 10 | 625 | 17 | 10 | 625 |
| 18 | 11 | 635.5 | 18 | 11 | 635.5 |
| 19 | 10 | 646 | 19 | 10 | 646 |
|  | Full-MS 350-1400 | / | 20 | 12 | 657 |
| 20 | 12 | 657 |  | Full-MS 350-1400 | / |
| 21 | 10 | 668 | 21 | 10 | 668 |
| 22 | 11 | 678.5 | 22 | 11 | 678.5 |
| 23 | 10 | 689 | 23 | 10 | 689 |
| 24 | 10 | 699 | 24 | 10 | 699 |
| 25 | 9 | 708.5 | 25 | 9 | 708.5 |
| 26 | 11 | 718.5 | 26 | 11 | 718.5 |
| 27 | 10 | 729 | 27 | 10 | 729 |
| 28 | 12 | 740 | 28 | 12 | 740 |
| 29 | 10 | 751 | 29 | 10 | 751 |
| 30 | 12 | 762 | 30 | 12 | 762 |
| 31 | 9 | 772.5 | 31 | 9 | 772.5 |
| 32 | 12 | 783 | 32 | 12 | 783 |
| 33 | 10 | 794 | 33 | 10 | 794 |
| 34 | 12 | 805 | 34 | 12 | 805 |
| 35 | 10 | 816 | 35 | 10 | 816 |
| 36 | 12 | 827 | 36 | 12 | 827 |
| 37 | 11 | 838.5 | 37 | 11 | 838.5 |
| 38 | 12 | 850 | 38 | 12 | 850 |
|  | Full-MS 350-1400 | / | 39 | 10 | 861 |
| 39 | 10 | 861 | 40 | 13 | 872.5 |
| 40 | 13 | 872.5 |  | Full-MS 350-1400 | / |
| 41 | 10 | 884 | 41 | 10 | 884 |
| 42 | 13 | 895.5 | 42 | 13 | 895.5 |
| 43 | 12 | 908 | 43 | 12 | 908 |
| 44 | 13 | 920.5 | 44 | 13 | 920.5 |
| 45 | 13 | 933.5 | 45 | 13 | 933.5 |
| 46 | 15 | 947.5 | 46 | 15 | 947.5 |
| 47 | 13 | 961.5 | 47 | 13 | 961.5 |
| 48 | 20 | 978 | 48 | 14 | 975 |
| 49 | 17 | 996.5 | 49 | 16 | 990 |
| 50 | 16 | 1013 | 50 | 16 | 1006 |
| 51 | 21 | 1031.5 | 51 | 19 | 1023.5 |
| 52 | 18 | 1051 | 52 | 18 | 1042 |
| 53 | 22 | 1071 | 53 | 20 | 1061 |
| 54 | 27 | 1095.5 | 54 | 25 | 1083.5 |
| 55 | 30 | 1124 | 55 | 28 | 1110 |
| 56 | 61 | 1169.5 | 56 | 30 | 1139 |
| 57 | 200 | 1300 | 57 | 35 | 1171.5 |
|  |  |  | 58 | 38 | 1208 |
|  |  |  | 59 | 40 | 1247 |
|  |  |  | 60 | 133 | 1333.5 |
